# Development of CD46 targeted alpha theranostics in prostate cancer using ^134^Ce/^225^Ac-Macropa-PEG_4_-YS5

**DOI:** 10.7150/thno.92742

**Published:** 2024-01-27

**Authors:** Kondapa Naidu Bobba, Anil P. Bidkar, Anju Wadhwa, Niranjan Meher, Suchi Drona, Alexandre M. Sorlin, Scott Bidlingmaier, Li Zhang, David M. Wilson, Emily Chan, Nancy Y. Greenland, Rahul Aggarwal, Henry F. VanBrocklin, Jiang He, Jonathan Chou, Youngho Seo, Bin Liu, Robert R. Flavell

**Affiliations:** 1 Department of Radiology and Biomedical Imaging, University of California, San Francisco, California 94143, United States.; 2 Department of Anesthesia, University of California, San Francisco, California 94110, United States.; 3 Department of Medicine and the Department of Epidemiology and Biostatistics, University of California, Berkeley, California, United States.; 4 UCSF Helen Diller Family Comprehensive Cancer Center, San Francisco, California 94143-0981, United States.; 5 Department of Pathology, University of California, San Francisco, California 94110, United States.; 6 Division of Hematology/Oncology, Department of Medicine, University of California, San Francisco, California, United States.; 7 Department of Radiology and Medical Imaging, University of Virginia, Charlottesville, Virginia, 22908, United States.; 8 Department of Pharmaceutical Chemistry, University of California, San Francisco, California 94158-2517, United States.

**Keywords:** targeted alpha therapy, PEG linkers, YS5 antibody, macropa, actinium-225, cerium-134, theranostics

## Abstract

**Rationale:** ^225^Ac, a long-lived α-emitter with a half-life of 9.92 days, has garnered significant attention as a therapeutic radionuclide when coupled with monoclonal antibodies and other targeting vectors. Nevertheless, its clinical utility has been hampered by potential off-target toxicity, a lack of optimized chelators for ^225^Ac, and limitations in radiolabeling methods. In a prior study evaluating the effectiveness of CD46-targeted radioimmunotherapy, we found great therapeutic efficacy but also significant toxicity at higher doses. To address these challenges, we have developed a radioimmunoconjugate called ^225^Ac-Macropa-PEG_4_-YS5, incorporating a stable PEGylated linker to maximize tumoral uptake and increase tumor-to-background ratios. Our research demonstrates that this conjugate exhibits greater anti-tumor efficacy while minimizing toxicity in prostate cancer 22Rv1 tumors.

**Methods:** We synthesized Macropa.NCS and Macropa-PEG_4/8_-TFP esters and prepared Macropa-PEG_0/4/8_-YS5 (with nearly ~1:1 ratio of macropa chelator to antibody YS5) as well as DOTA-YS5 conjugates. These conjugates were then radiolabeled with ^225^Ac in a 2 M NH_4_OAc solution at 30 °C, followed by purification using YM30K centrifugal purification. Subsequently, we conducted biodistribution studies and evaluated antitumor activity in nude mice (nu/nu) bearing prostate 22Rv1 xenografts in both single-dose and fractionated dosing studies. Micro-PET imaging studies were performed with ^134^Ce-Macropa-PEG_0/4/8_-YS5 in 22Rv1 xenografts for 7 days. Toxicity studies were also performed in healthy athymic nude mice.

**Results:** As expected, we achieved a >95% radiochemical yield when labeling Macropa-PEG_0/4/8_-YS5 with ^225^Ac, regardless of the chelator ratios (ranging from 1 to 7.76 per YS5 antibody). The isolated yield exceeded 60% after purification. Such high conversions were not observed with the DOTA-YS5 conjugate, even at a higher ratio of 8.5 chelators per antibody (RCY of 83%, an isolated yield of 40%). Biodistribution analysis at 7 days post-injection revealed higher tumor uptake for the ^225^Ac-Macropa-PEG_4_-YS5 (82.82 ± 38.27 %ID/g) compared to other conjugates, namely ^225^Ac-Macropa-PEG_0/8_-YS5 (38.2 ± 14.4/36.39 ± 12.4 %ID/g) and ^225^Ac-DOTA-YS5 (29.35 ± 7.76 %ID/g). The PET Imaging of ^134^Ce-Macropa-PEG_0/4/8_-YS5 conjugates resulted in a high tumor uptake, and tumor to background ratios. In terms of antitumor activity, ^225^Ac-Macropa-PEG_4_-YS5 exhibited a substantial response, leading to prolonged survival compared to ^225^Ac-DOTA-YS5, particularly when administered at 4.625 kBq doses, in single or fractionated dose regimens. Chronic toxicity studies observed mild to moderate renal toxicity at 4.625 and 9.25 kBq doses.

**Conclusions:** Our study highlights the promise of ^225^Ac-Macropa-PEG_4_-YS5 for targeted alpha particle therapy. The ^225^Ac-Macropa-PEG_4_-YS5 conjugate demonstrates improved biodistribution, reduced off-target binding, and enhanced therapeutic efficacy, particularly at lower doses, compared to ^225^Ac-DOTA-YS5. Incorporating theranostic ^134^Ce PET imaging further enhances the versatility of macropa-PEG conjugates, offering a more effective and safer approach to cancer treatment. Overall, this methodology has a high potential for broader clinical applications.

## Introduction

Radiopharmaceutical therapy is a rapidly growing therapeutic modality for treating cancer. β-emitting radionuclides, including ^177^Lu, ^90^Y, and ^131^I are commonly used in targeted radiotherapies 1-3. Notably, the recent approval by the Food and Drug Administration of [^223^Ra]RaCl_2_ for treating bone metastases in metastatic castration-resistant prostate cancer (mCRPC), marked a significant milestone, paving the way for targeted alpha therapies in cancer 4,5. Targeted alpha therapy (TAT) is facilitated through vectors such as antibodies, small molecules, or peptides, and can precisely deliver concentrated doses of radiation to malignant cells, inducing their demise 6,7. Alpha-emitters hold a distinct advantage over β-emitters due to their heightened linear energy transfer (LET) of 100 keV/mm as opposed to 1-2 keV/mm, resulting in the more pronounced generation of free radicals and lethal DNA double-strand breaks 8,9.

The α-emitting radionuclide ^225^Ac produces 4 α and 2 β^-^ particles as it decays and has attracted particular attention for radiopharmaceutical therapy 10. For example, targeted alpha therapy using small molecule inhibitors of prostate-specific membrane antigen (PSMA) has demonstrated efficacy in small clinical studies 11-13. In this context, the ^225^Ac-PSMA-617 conjugate exhibited greater efficacy in patients with metastatic castration-resistant prostate cancer, even in cases resistant to ^177^Lu-PSMA-617 treatment 7. Similarly,^225^Ac-DOTATATE, a radiolabeled somatostatin analog, demonstrated therapeutic efficacy in ^177^Lu-DOTATATE refractory patients 14. ^225^Ac has also been employed for radioimmunotherapy. For example, a CD33-targeted radioimmunoconjugate utilizing the HuM195 antibody (Lintuzumab) has displayed promise in phase I/II clinical trials for acute myeloid leukemia treatment 15. Recently, favorable therapeutic and toxicity results were reported in a clinical study for ^225^Ac-J591, a PSMA targeting antibody 16. These findings underscore the potential of targeted ^225^Ac alpha radiopharmaceutical therapy in cancer 17.

In addition to modality, target selection is critical for successful cancer radioimmunotherapy. In our search of novel tumor cell surface targets, we selected human antibody phage display libraries on tumor cells and patient samples 18-20, identified novel tumor binding antibodies, and used them to pull down target antigens for identification by mass spectrometry. Using this method, we identified CD46 as a novel target in prostate cancer 21 and multiple myeloma 22. We identified a novel human antibody YS5 that binds to a tumor-selective CD46 epitope with negligible binding to normal healthy tissues except for placenta trophoblasts and to a lesser degree the prostate epithelium, opening the door for therapeutic targeting of CD46 that is overexpressed in prostate cancer and a variety of other malignancies. The YS5 antibody was used to develop an antibody-drug conjugate which displayed potent anti-tumor activity both in vitro and in vivo using prostate cancer cell line xenograft models and was found to be well tolerated in non-human primate studies 21. This promising conjugate has progressed to clinical trials, currently being evaluated in two multiple center phase I single-agent trials (NCT03575819 in prostate cancer and NCT03650491 in multiple myeloma), and a phase I/II combination trial (NCT03575819) 23. We further developed a paired ImmunoPET imaging agent, ^89^Zr-DFO-YS5, as a companion diagnostic. This agent has demonstrated accumulation in a variety of prostate cancer and multiple myeloma preclinical models 24. Building upon this success, studies of this PET agent in patients with mCRPC (NCT05245006) and multiple myeloma (NCT05892393) are currently underway. Recently, we applied this antibody for targeted alpha therapy using ^225^Ac-DOTA-YS5 25,26 and ^212^Pb-TCMC-YS5 27, showing longer survival rates in preclinical prostate cancer and multiple myeloma tumor models. Taken together, these preclinical and clinical studies demonstrate great promise for CD46-directed theranostics and targeted alpha therapy for cancer treatment.

While ^225^Ac-based radiotherapy holds high potential, some challenges limit its routine implementation. Notably, irradiating healthy tissues may lead to toxicity, for example, xerostomia following ^225^Ac-PSMA-617 therapy 28. Similarly, redistribution of daughter nuclei can lead to non-specific radiopharmaceutical accumulation, resulting in off-target toxicities (e.g., in the kidney) 29. Consequently, a balance must be struck between antitumor activity and toxicity. These limitations emphasize the pressing need for innovative strategies to visualize and mitigate the potential toxicity of ^225^Ac-based radioimmunotherapy while optimizing the antitumor accumulation of targeted agents.

In recent years, linker chemistry methodology has made notable enhancement of antibody-drug conjugate targeted delivery, improving their pharmacokinetics by reducing off-target toxicity via the slow release of cytotoxic payloads at targeted sites 30. Recently, radiolabeled antibodies with short PEG linkers were shown to accelerate blood clearance with concomitant higher tumor uptake, leading to improved tumor-to-blood and organ ratios 31. Taken together, prior and ongoing work suggests that inserting an appropriately selected linker between an antibody and cytotoxic payload may reduce the off-target toxicity.

The recently described macropa chelator exhibits superior ^225^Ac radiolabeling kinetics and efficiency in comparison to the commonly used DOTA 32. Notably, we have recently employed the macropa chelator in conjunction with ^134^Ce/La to act as a surrogate for PET imaging in the context of alpha therapeutics. ^134^Ce with a half-life (t_1/2_ = 3.2 days) was produced and supplied by the Department of Energy isotope (DOE) program. This isotope undergoes decay to ^134^La (t_1/2_ = 6.45 min) via electron capture. Subsequently, it further decays to stable ^134^Ba through positron emission (63% β^+^; endpoint energy, 2.69 MeV) 33. This strategy has particular promise for the development of theranostics, owing to the chemical similarity between Ce^3+^ and Ac^3+^, as well as the high fraction of positrons emitted by the daughter isotope ^134^La 34. Notably, ^134^Ce allows for direct labeling of the macropa chelator for imaging, which is not possible using more commonly used imaging isotopes such as ^68^Ga, ^89^Zr or ^64^Cu.

In this work, we combine the use of novel linker technology with the high-affinity macropa chelator to conjugate the therapeutic radioisotope ^225^Ac with the specific targeting antibody YS5 for the treatment of prostate cancer. Additionally, the radiotherapeutic distribution may be visualized by ^134^Ce/^134^La immunoPET imaging. The therapeutic efficacy of the resulting conjugate, ^225^Ac-Macropa-PEG_4_-YS5 was enhanced, when compared against the previously described ^225^Ac-DOTA-YS5 conjugate prepared by conventional methodology. This chelator and linker combination could be used as a general strategy to increase the therapeutic efficacy of targeted alpha therapy.

## Methods

### Antibody conjugation

The approach employed for antibody conjugation was adapted from a previously published protocol 24. In this method, YS5 (1 mg) in HEPES buffer underwent buffer exchange through three cycles with 0.1 mol/L Na_2_CO_3_-NaHCO_3_ buffer (200 μL) at pH 9.0. This exchange was performed using a YM30K MW centrifugal filter unit (Millipore, MA, USA). Macropa-NCS and Macropa-PEG_4/8_-TFP esters, which had been freshly prepared and were stored at -20°C, were dissolved in DMSO (1 mg/50 μL). Subsequently, the YS5 antibody was subjected to incubation with different equivalents of Macropa-NCS and Macropa-PEG_4/8_-TFP esters at 37 °C for a duration of 2 h. Following the conjugation process, the mixture was purified using PD10 gel column filtration from GE Healthcare. Elution was achieved using 0.25 M NaOAc (pH = 6) as the mobile phase. The resulting compounds were stored at -20 °C and used without further modifications for subsequent radiolabeling and analysis purposes.

### Radiolabeling with various amounts of Macropa-PEG_0/4/8_-YS5 conjugates versus Ac-225

A standard one-step process was used for radiolabeling. The Ac-225 was received from Oak Ridge National Laboratory (ORNL) and it was produced through a Th-229 generator 35. The solid ^225^Ac-nitrate (37 MBq) was dissolved in 0.2 M HCl (100 µL). An aliquot of 1 µL (185 kBq) was added to a vail containing L-ascorbic acid (150 g/L; 20 µL), and the corresponding antibody construct (2.5, 5, 10, 20 µg), 2 M ammonium acetate buffer (pH = 5.8, 50 µL). The reaction mixture was placed in a thermomixer (500 rpm) and incubated at 30.0 °C for 30 min. For DOTA-YS5, the reaction was performed at 40 ^o^C for 2 h. The radiochemical yields were measured by spotting a small aliquot on a strip of instant thin-layer chromatography using ITLC-SG, eluted with 10 mM ethylenediaminetetraacetic acid (EDTA), pH = 5.5.

### Radiolabeling of Macropa-PEG_0/4/8_-YS5 conjugates

An aliquot of 5 µL (925 kBq) of Ac-225 was added to a vail containing L-ascorbic acid (150 g/L; 20 µL), and the corresponding antibody construct (100 µg), 2 M ammonium acetate buffer (pH = 5.8, 50 µL). The reaction mixture was placed in a thermomixer (500 rpm) and incubated at 30.0 °C for 30 min. For DOTA-YS5, the reaction was performed at 40 ^o^C for 2 h. The radiochemical yields were measured by spotting a small aliquot on a strip of instant thin-layer chromatography using iTLC-SG, eluted with 10 mM EDTA, pH = 5.5. The radioimmunoconjugates were subjected to buffer exchange with 0.9% saline (3 x 400 µL) using centrifugal filtration (Fisher Scientific Accuspin Micro 17R centrifuge) with a YM30K molecular weight cutoff. Final compounds demonstrated >95% radiochemical purity.

### Stability studies

^225^Ac-Macropa-PEG_0/4/8_-YS5 (~555-925 kBq, Radiochemical purity >95% after centrifugal filtration purification, 55-65 µL) were diluted with 500 µL of either saline or human serum at 37 ^o^C. At the indicated time points, 5-10 µL aliquots were analyzed by radio-iTLC-SG using 10 mM EDTA, pH = 5.5 as an eluent. All radio-iTLC's were scanned after 24 h equilibrium was reached.

### Stability studies by size exclusion chromatography (SEC)

^225^Ac-Macropa-PEG_0/4/8_-YS5 (~925 kBq, radiochemical purity > 95% after centrifugal filtration purification 10-15 µL) were diluted with 2 mL of 1% human serum albumin (Sigma-Aldrich, Cat. no. 12666) in saline. An aliquot of ~70-95 µL was injected in SEC at days 0, 3, 5, and 7 and collected 1 mL fractions until 30 min. The collected fractions were counted on the hidex gamma counter after 24 h secular equilibrium and then plotted time versus counts per minute (cpm). Similarly, radio-iTLC-SG was also checked at the same time points, and TLC's were scanned after 24 h secular equilibrium was reached.

### Cell culture

The 22Rv1 prostate cancer cells (RRID: CVCL_1045) were sourced from the American type culture collection (ATCC). These cells were grown in a CO_2_ incubator using RPMI medium supplemented with 10% fetal bovine serum and a 1% penicillin and streptomycin antibiotic solution. The STR phenotyping conducted at ATCC confirmed the cell line's authenticity. The 22Rv1 cell lines were also screened for mycoplasma contamination using the mycoalert mycoplasma detection kit, yielding negative results.

### Cell Binding assay for ^225^Ac-Macropa-PEG_4_-YS5 in 22Rv1 cells

A binding assay was conducted to validate the binding of ^225^Ac-Macropa-PEG_4_-YS5 following radiolabeling. In this experiment, ^225^Ac-Macropa-PEG_4_-YS5 at a concentration of 7 nM was incubated with 22Rv1 cells (1 million cells per tube) in triplicate for 1 h. To assess binding specificity, blocking samples were prepared by adding a 10-fold excess of cold YS5. Additionally, 1% milk protein was introduced to the tubes to prevent the non-specific binding of ^225^Ac-Macropa-PEG_4_-YS5. After incubation, the cells were centrifuged at 500 x g and washed twice with saline to remove any unbound ^225^Ac-Macropa-PEG_4_-YS5. The quantification ^225^Ac-Macropa-PEG_4_-YS5 bound to 22Rv1 cells was counted on the hidex gamma counter with a gamma energy window set between 25 and 2000 keV after reaching a 24 h secular equilibrium.

### The in-vitro cell-killing activity of ^225^Ac-Macropa-PEG_4_-YS5

To assess the impact of ^225^Ac-Macropa-PEG_4_-YS5 and non-targeted ^225^Ac-DOTA and Macropa on cell viability, 22Rv1 cells were cultivated in 96-well plates at a density of 2,000 cells per well. The cells were treated with varying concentrations of ^225^Ac-Macropa-PEG_4_-YS5 (ranging from 3.7 x 10^-7^ to 3.7 kBq) for 96 h. After the treatment, cell viability was measured using the cell titer glo reagent (Fisher scientific, Mfr. No. Promega G7570) per the manufacturer's instructions. The % cell viability values were fitted into a sigmoidal dose-response curve using the GraphPad Prism program to analyze the data.

### Xenograft models

The animals in this study were handled in compliance with the institutional animal care and use committee-approved guidelines at the laboratory animal resource center (LARC) at the University of California San Francisco. Male athymic homozygous nude mice aged 5-6 weeks (Strain: 002019, Homozygous) from Jackson laboratories were subcutaneously xenografted with 22Rv1 cells (2.5 x 10^6^) in a 1:1 mixture of matrigel and PBS buffer. Biodistribution and therapy studies were performed when the tumors reached a size of ~100-150 mm^3^.

### ^134^Ce/La radio labeling of nearly 1:1 ratio of Macropa-PEG_0/4/8_-YS5

To Macropa-PEG_0/4/8_-YS5 (358 μg; 1:1.5 total metal to YS5 molar ratio, 7.3-7.73 mg/mL) was added an aliquot of ^134^CeCl_3_ (56 μL, 55 MBq) in 1 M NH_4_OAc (200 µL, pH = 8.0). The mixture was incubated at 25 ^o^C for 1 h. The radiolabeling progress was monitored by instant thin layer chromatography (TLC) on Varian iTLC-SG strips using 10 mM EDTA, pH = 5.5 as an eluent. The radiolabeling conversions were 100% for PEG_0_, 96.97% for PEG_4_, and 91.45% for PEG_8_. The reaction mixture was purified over PD10 column gel filtration eluting with 0.9% saline solution and found to be ~100% radiochemical purity for all the conjugates by radio iTLC using 10 mM EDTA, pH~5.5. The isolated yield for ^134^Ce-Macropa-PEG_0/4/8_-YS5 (41.92, 48.1, 48.5 MBq) with a molar activity of 17.4, 20.16, and 20.53 GBq/µmol.

### Ex-vivo biodistribution study in 22Rv1 xenografts

The ex-vivo biodistribution studies were conducted on healthy athymic nude mice bearing subcutaneous 22Rv1 xenografts, which were approximately ~100-150 mm^3^ in size. The radioimmunoconjugates ^225^Ac-Macropa-PEG_0/4/8_-YS5 (18.5 kBq in 100 µL of 0.9% NaCl solution) with different chelator ratios per antibody were administered via tail vein. the mice (n = 3 or 4) were euthanized and tissues of interest were collected at an appropriate time (day 1, 2, 4, and 7) post-injection. Each sample was counted for up to 1 min using a hidex automatic gamma counter set up to a 25 to 2000 keV energy window. The counts were measured 24 h after collection when secular equilibrium was reached. The results were expressed as the percentage of injected dose (%ID) and determined by counting standards prepared from the formulated radiotracer and the tissue samples. The counts from each sample were decay corrected and background corrected, and the count in each sample was converted to %ID/g by comparison against standards of known reactivity.

### Dosimetry

Conducting dosimetry calculations involved using the time-integrated activity coefficients and employing curve-fitting within the EXM module of OLINDA/EXM Version 1.1. The digital mouse phantom in OLINDA Version 2.0 was utilized for this purpose. Systematically organizing the biodistribution study data allowed the establishment of time and the percentage of injected activity for each organ and tumor. These numerical values were then employed as inputs to derive time-integrated activity coefficients. Subsequently, the equivalent dose (in Sv) was computed as the product of absorbed dose (in Gy) and radiation weighting factors.

### In vivo therapy

The mice were randomized into four therapy groups and one control group with eight animals per group. After randomization, the mice were administered varying doses of the radioimmunoconjugates ^225^Ac-Macropa-PEG_4_-YS5 and DOTA-YS5 (4.625 and 9.25 kBq) in 0.9% sterile saline (~100 mL) intravenously and the control group was administered ~100 μL of saline. Tumor volumes were measured twice weekly until mice reached a set endpoint: decreased body condition score, severe petechiae, >20% weight loss, or tumor volume exceeding 2,000 mm^3^.

### Chronic Toxicity study in nude mice

The toxicity of ^225^Ac-Macropa-PEG_4_-YS5 was investigated in healthy nude mice aged 5-6 weeks obtained from Jackson laboratories (Strain: 002019, Homozygous). For the chronic toxicity assessment, the mice were divided into groups (n = 5 mice per group) and injected with either 4.625 or 9.25 kBq of ^225^Ac-Macropa-PEG_4_-YS5, while another group received a vehicle saline injection. The mice were closely monitored throughout the experiment (twice weekly), and their body weights were measured twice a week for 120 days. After the study, comprehensive blood tests were performed, including a complete blood count and laboratory tests to evaluate organ function. A cardiac puncture procedure was carried out to collect the blood samples at the time of sacrifice, and the blood was collected in EDTA-coated tubes to prevent coagulation and study blood cell counts. Additionally, serum samples were obtained by allowing the blood-containing vials to sit at 4 °C for 30 min to separate the serum from the clotted blood. The serum samples were then separated from the clots by centrifugation at 10,000 x g for 10 min at 4 °C. The blood and serum samples were sent to the comparative pathology laboratory at the University of California Davis School of Veterinary Medicine for analysis. The laboratory conducted blood cell counts and organ function tests to assess the potential toxicity of ^225^Ac-Macropa-PEG_4_-YS5.

### Histology of the tissue sections

After euthanasia, various organs, including the liver, kidney, lungs, spleen, heart, and bone were extracted from all mice and fixed in 10% neutral buffered formalin. Subsequently, routine histologic analysis was carried out to examine the microscopic features of the tissues. For Hematoxylin and Eosin (H&E) staining, the tissues were fixed in formalin, processed through an ethanol gradient (30% to 70%), and embedded in paraffin. Tissue sections with a thickness of 4 µm were prepared for H&E staining. Additionally, as necessary, a subset of organs underwent staining using periodic acid-schiff (PAS) and trichrome staining. To assess ^225^Ac-Macropa-PEG_4_-YS5 related toxicity, histologic changes in the treated mice were compared to those observed in the saline control mice.

### Analysis of CD46 expression in correlation with the ^225^Ac-Macropa-PEG_4_-YS5 uptake and DNA damage

To correlate the expression of the CD46, ^225^Ac-Macropa-PEG_4_-YS5 distribution and the DNA damage events, tumors from a cohort of mice injected with ^225^Ac-Macropa-PEG_4_-YS5 were euthanized on days 1, 2, 4, and 7 (n = 3 mice for each time point). Tumor sections from these four time points were subjected to digital autoradiography, CD46 IF, phospho-H2AX, and H & E staining. For digital autoradiography, tissue sections were placed on an iQID digital camera, set to acquire decay events at 2 volts. For IF staining, tissue sections were fixed with acetone (-20 ^o^C), stained with anti-CD46 antibody (Abcam, Cat. no. ab231984, 1: 300 dilutions) and anti-gamma phospho-H2Ax (Alexa Fluor® 647 conjugate, Cat. no. 80312, 1:250 dilutions) antibodies; while nuclei were stained with DAPI (Thermo Scientific, Cat no. 62248, 1:1000 dilutions from 1 mg/mL). Secondary staining was carried out with an anti-rabbit secondary antibody (Alexa Fluor® 647 conjugate, Cell signaling technology, Cat. no. 4414S, 1:1000 dilutions). For histology, tissues were stained using an H & E staining kit (Abcam, Cat. no. ab245880).

### Statistical Analysis

All data were expressed as mean ± SD. Data were analyzed using GraphPad Prism 8, and a P value < 0.05 was considered statistically significant. Two-way ANOVA was used for calculating the biodistribution, tumor-to-background ratios. The log-rank sum test was used for survival analysis. One-way ANOVA was used to calculate the kidney, liver function tests, and blood analysis.

## Results

### Synthesis of Macropa-PEG_0/4/8_-YS5 conjugates

**Figure 1:**
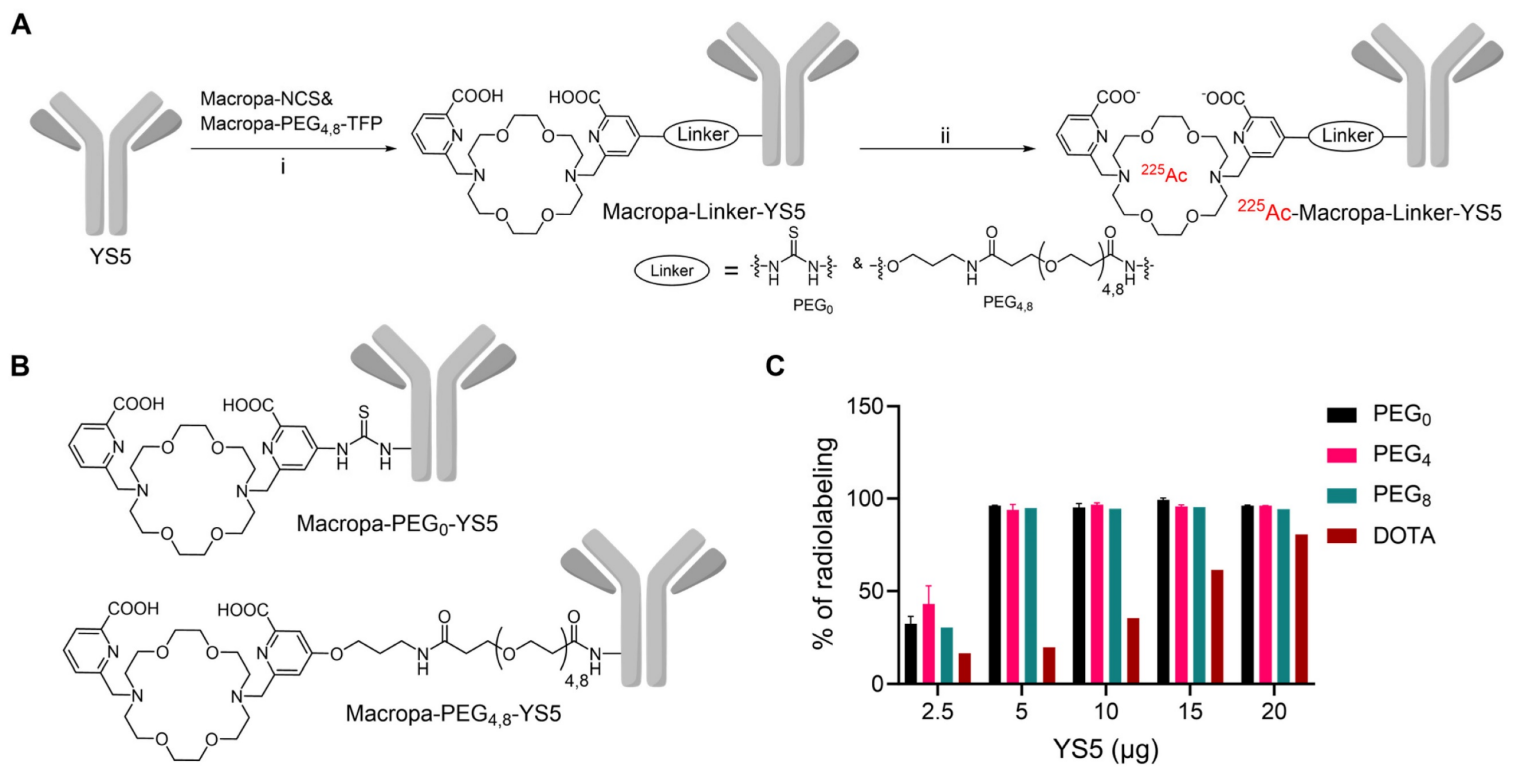
Synthesis and radiolabeling of Macropa-PEG_0/4/8_-YS5 conjugates A) Bioconjugation of YS5 with Macropa-NCS and Macropa-PEG_4/8_-TFP ester followed by radiolabeling with ^225^Ac(NO_3_)_3_ i) 0.1 M Na_2_CO_3_-Na_2_HCO_3_ buffer, pH = 9.0, 37 ^o^C, 2 h followed by PD10 column purification and ii) ^225^Ac(NO_3_)_3_ in 0.2 M HCl, 150 mg/mL L-Ascorbic acid, 2 M NH_4_OAc, pH = 5.8 followed by centrifugal filtration. B) Structures of Macropa-PEG_0/4/8_-YS5 C) Radiochemical yields for ^225^Ac-Macropa-PEG_0_-YS5, ^225^Ac-Macropa-PEG_4_-YS5, ^225^Ac-Macropa-PEG_8_-YS5 and ^225^Ac-DOTA-YS5 (In the figure legend denoted as a PEG_0_, PEG_4_, PEG_8_, and DOTA). n = 2 reactions for PEG_0_, PEG_4_ and n = 1 reactions for PEG_8_, DOTA.

Macropa-PEG_8_-TFP ester was synthesized by reacting the intermediate 1 in the presence of DIPEA in DMF followed by prep HPLC purification in 34% yield (Reaction scheme S1 and Figure S1-S3). Macropa NCS was synthesized according to the reported literature 32, and Macropa-PEG_4_-TFP was synthesized as per our prior publication 33. With these derivatives in hand, the bifunctional chelators were conjugated to lysine residues on the antibody YS5 (Figure 1A, B) 24. As the bioconjugation on YS5 with chelators is non-specific, a greater number of chelators per antibody was obtained during the initial conditions. By varying different molar ratios, we finally managed to have a nearly 1:1 ratio of macropa chelator per antibody YS5, as determined by MALDI-TOF MS (Table S1 and Figure S4-S8). Two different variants with low (~1:1 ratios) and high copy numbers (>2.5 chelators per YS5) of the chelator relative to the antibody (0.56 and 4.5 for Macropa-PEG_0_-YS5, 0.91 and 2.6 for Macropa-PEG_4_-YS5, and 0.96 and 7.7 for Macropa-PEG_8_-YS5, respectively), were advanced for radiolabeling and biodistribution studies. DOTA-YS5 was synthesized as previously described, with an average of 8.7 chelators per antibody 25.

### Radiolabeling, In vitro stability studies, and immunoreactivity assay

In order to develop an optimized radiosynthesis, we compared the ^225^Ac radiolabeling efficiency of the DOTA and macropa conjugates (~1:1 ratio per antibody YS5) utilizing various amounts of the precursor ranging from 2.5 - 20 µg (concentration of 7.5 - 33 mg/mL, Figure 1C and Figure S9-12). Notably, radiochemical yields (RCY) of >94% were found for all macropa conjugates at 5 µg of protein, starting from 185 kBq activity. In contrast, with 5 µg of DOTA-YS5, 16.4% RCY was obtained, and increasing DOTA-YS5 to 20 µg gave only 79% RCY. These results are consistent with prior studies demonstrating improved ^225^Ac radiolabeling using macropa conjugates when compared to DOTA conjugates 32.

These optimized conditions using a 1:4 metal-to-antibody were scaled up for subsequent *in vitro* and *in vivo* experiments, utilizing conjugates with both low and higher copy numbers of chelators per antibody (Figure S13). Excellent radiochemical yields were seen for all macropa conjugates, regardless of low or high chelator derivatization (94.8 ± 2.5 - 97.7 ± 1.6% as measured by radio iTLC, and 33.1 ± 9.0 - 80 ± 0.0% isolated yield). All final compounds were >96% pure, with specific activities ranging from 2.96 ± 0.02 to 7.4 MBq/mg. SEC revealed no evidence of aggregation of any conjugates (Figure S14). The target binding fraction for all the radioimmunoconjugates was analyzed using a magnetic bead-based radioligand assay. A target binding fraction of 88.0 ± 16.7% for ^225^Ac-Macropa-PEG_0_-YS5, 88.8 ± 7.3% for ^225^Ac-Macropa-PEG_4_-YS5, and 99.4 ± 18.7% ^225^Ac-Macropa-PEG_8_-YS5 was observed (n = 3). While the more highly labeled PEG_0_ and PEG_4_ derivatives demonstrated preserved immunoreactivity, the conjugate with 7.7 chelators per antibody conjugate ^225^Ac-Macropa-PEG_8_(7.7)-YS5 showed a decreased binding fraction of 78.3 ± 1.9% (Figure S15). These data demonstrate that the Ac-225 labeled conjugates retain immunoreactivity after bioconjugation and radiolabeling steps.

We tested the stability of the Ac-225 labeled Macropa-PEG_0/4/8_-YS5 conjugates in two different methods. In the first method, the radioimmunoconjugates were incubated in human serum at 37 ^o^C for 21 days, and stability was monitored by radio iTLC. As shown in Figure S17A, B, ^225^Ac-Macropa-PEG_4_-YS5_,_ and ^225^Ac-Macropa-PEG_8_-YS5 showed 100% complex stability in human serum over the time course. In contrast, the ^225^Ac-Macropa-PEG_0_-YS5 derivative demonstrated a complex pattern with multiple peaks (R_f_) on TLC at all time points (Figure S17C). To evaluate further, we tested the stability of the radiopharmaceuticals in 1% HAS in saline using SEC followed by radio iTLC. The results indicated that ^225^Ac-Macropa-PEG_4/8_-YS5 exhibited remarkable stability, and was fully intact until 7 days in both SEC and radio iTLC (Figure S18,19). However, the ^225^Ac-Macropa-PEG_0_-YS5 conjugate displayed multiple additional peaks at 13 min and 14-15 min in addition to the expected 10-11 min. peak, starting from day 3 with 29.4% intact to day 7 with 38.2% intact (Figure S20A & Table S3). In contrast, the radio iTLC of ^225^Ac-Macropa-PEG_0_-YS5 conjugate showed 100% complex stability until 7 days. This discrepancy may be explained by the generation of metabolic fragments which cannot be separated by iTLC, but are well resolved by SEC (Figure S20B). ^225^Ac-DOTA-YS5 demonstrated relatively good stability (85% intact) over 7 days with slow degradation compared with ^225^Ac-Macropa-PEG_0_-YS5 conjugate 38.2% intact by SEC (Figure S21A). The radio iTLC showed almost 100% complex stability (Figure S21B). Prior reports have indicated that Macropa NCS-based conjugates have demonstrated high stability, conflicting with our results 32. This apparent difference in findings may be due to the formation of hypochlorite ions upon radiation with a thiourea bond between the chelator macropa and antibody YS5 36. In contrast, ^225^Ac-Macropa-PEG_4/8_-YS5 exhibited high in vitro stability, likely attributed to the more stable amide linkage 37. Overall, these results demonstrated excellent stability for the ^225^Ac-Macropa-PEG_4/8_-YS5 conjugates compared to ^225^Ac-DOTA-YS5 and ^225^Ac-Macropa-PEG_0_-YS5.

### In vivo biodistribution, dosimetry, and metabolic analysis

**Figure 2:**
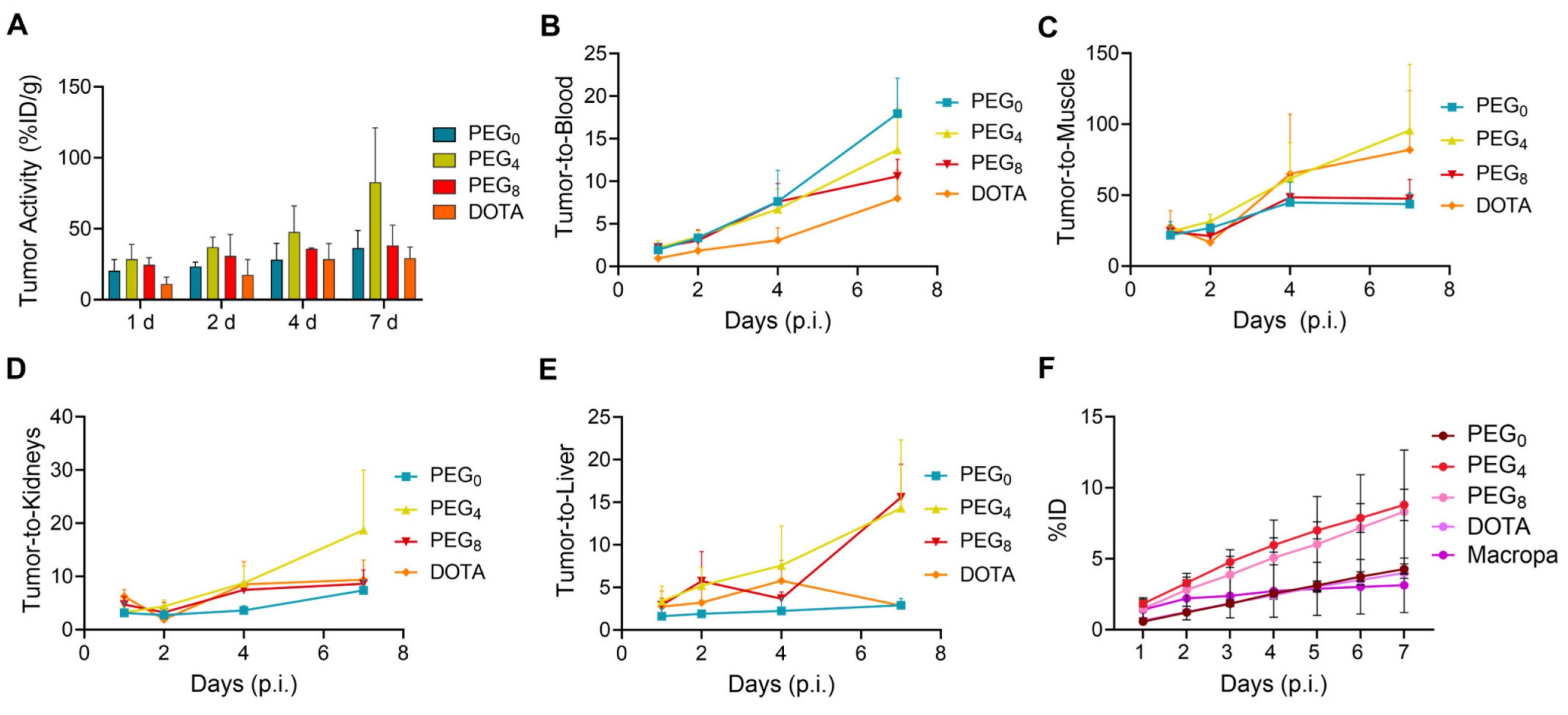
^225^Ac-Macropa-PEG_4_-YS5 demonstrates increased tumor uptake with improved tumor-to-background ratios and clearance profiles compared to other conjugates. A) Tumor uptakes from the ex-vivo biodistribution analysis of ^225^Ac-Macropa-PEG_0/4/8_-YS5 and ^225^Ac-DOTA-YS5 in 22Rv1 xenografts after tail vein injection at day 1, 2, 4, and 7. B) Tumor to blood C) Tumor to muscle D) Tumor to kidney E) Tumor to liver ratios derived from ex vivo biodistribution analysis. F) Metabolic analysis of ^225^Ac-Macropa-PEG_0/4/8_-YS5 and ^225^Ac-DOTA-YS5 in healthy nude mice over 7 days, %ID in urine. ^225^Ac-DOTA-YS5 biodistribution data was reproduced. Adapted with permission from 22, copyright 2023 American Association for Cancer Research.

The biodistribution of the ^225^Ac conjugates was evaluated in the subcutaneous 22Rv1 prostate cancer xenograft model, to select a lead compound for therapeutic evaluation. The biodistribution was compared using nearly ~1:1 ratios of chelator per antibody YS5 (only variation in the linker length). In order to further evaluate the role of chelator modification, we compared with >2.5 chelators per antibody conjugate with respective nearly 1:1 chelator ratio conjugate. As expected, tumor uptake for all the conjugates gradually increased over time, with concomitant clearance from blood and non-target organs (Figure S22A-D and Table S4-6). Higher tumor uptake for ^225^Ac-Macropa-PEG_4_-YS5 was observed at 7 day post injection (82.8 ± 38.3 %ID/g) in comparison with non-PEGylated conjugates ^225^Ac-Macropa-PEG_0_-YS5 (38.2 ± 14.4 %ID/g), ^225^Ac-DOTA-YS5 (29.3 ± 7.7 %ID/g). and PEGylated conjugates ^225^Ac-Macropa-PEG_8_-YS5 (36.4 ± 12.4 %ID/g) (Figure 2A). Parallel biodistribution studies using the YS5 macropa derivatives with higher chelator substitution demonstrated comparatively reduced tumor uptake (Figure S23) when compared against their less modified counterparts (^225^Ac-Macropa-PEG_0_-YS5: 18.5 ± 7.2 *vs.* 36.4 ± 12.4, ^225^Ac-Macropa-PEG_4_-YS5: 34.7 ± 9.1 *vs.* 82.8 ± 38.3 and ^225^Ac-Macropa-PEG_8_-YS5: 18.51 ± 5.55 vs 38.15 ± 14.41 %ID/g respectively) at 7 day post-injection. These data confirm superior tumor targeting for compounds with lower chelator loading.

Notably, clearance of activity from the blood was faster for ^225^Ac-Macropa-PEG_0_-YS5 (2.01 ± 0.43 %ID/g), and ^225^Ac-Macropa-PEG_8_-YS5 (3.86 ± 1.33 %ID/g) in comparison with ^225^Ac-Macropa-PEG_4_-YS5 (5.87 ± 0.83 %ID/g), at 7 days post-injection. Faster liver clearance was observed with the PEGylated linker derivatives, with 6.04 ± 1.72, 2.56 ± 1.19 %ID/g at day 7 for ^225^Ac-Macropa-PEG_4_-YS5 and ^225^Ac-Macropa-PEG_8_-YS5 compared to non-PEGylated conjugates ^225^Ac-Macropa-PEG_0_-YS5 (12.97 ± 4.5 %ID/g) and ^225^Ac-DOTA-YS5 (10.32 ± 2.96 %ID/g), respectively (Figure S22). Higher uptake with statistical significance was seen in the tumor for ^225^Ac-Macropa-PEG_4_-YS5 derivative at all time points when compared against the other derivatives, with the exception of ^225^Ac-Macropa-PEG_8_-YS5 at the day 1 and 2 time points only (Table S7).

When comparing tumor uptake ratios to healthy organs, the macropa derivatives showed generally improved characteristics compared to ^225^Ac-DOTA-YS5, with promising findings for ^225^Ac-Macropa-PEG_4_-YS5. Higher tumor-to-blood (13.70 ± 4.93 %ID/g), tumor-to-liver (14.3 ± 8.1 %ID/g), tumor-to-muscle (95.7 ± 46.4 %ID/g), and tumor-to-kidney (18.7 ± 11.3 %ID/g) were observed for ^225^Ac-Macropa-PEG_4_-YS5 in comparison with other conjugates at 7 days post-injection except for ^225^Ac-Macropa-PEG_0_-YS5 tumor-to-blood ratios (17.9 ± 4.2 %ID/g)(Figure 2B-E and Table S8, S9).

Prior to conducting the radiotherapy experiments, dosimetry calculations were undertaken to guide dose selection. Dosimetric analysis was performed assuming that ^225^Ac is the dominant emitter and primary contributor to the therapeutic effect of alpha particles. Daughter isotope redistribution was not accounted for in these calculations. The ^225^Ac-Macropa-PEG_4_-YS5 conjugate delivered a higher dose of approximately 29.07 Sv when compared to ^225^Ac-Macropa-PEG_0_-YS5 (10.42 Sv), and ^225^Ac-Macropa-PEG_8_-YS5 (21.55 Sv). As anticipated, the kidney had a higher absorbed dose than non-targeted organs (Table S10). As observed in our previous study with ^225^Ac-DOTA-YS5 and in other prior published studies 25, the higher dose absorbed in the kidney results from the redistribution of ^213^Bi after decay due to the nuclear recoil effect. Consequently, the kidney is the critical dose-limiting organ in CD46-targeted ^225^Ac-based radioligand therapies. These biodistribution studies confirmed that the macropa derivatives with lower chelator-to-antibody ratio demonstrated promise for therapeutic evaluation, with particularly high tumoral uptake as well as tumor-to-background, and tumor-to-kidney ratio observed for ^225^Ac-Macropa-PEG_4_-YS5; While the dosimetry estimate favored the PEG_8_ derivative, the PEG_4_ derivative was favored for the 7 day time point based on the tumor to kidney ratio. Given the imprecision of the dosimetry measurement, we felt this was best given the high tumor uptake.

To further investigate the role of the linkers and conjugation chemistry in the biodistribution of the ^225^Ac, metabolic excretion studies were performed. The conjugates ^225^Ac-Macropa-PEG_0_-YS5 (4.26 ± 0.36%) and ^225^Ac-DOTA-YS5 (3.99 ± 0.38%) were eliminated slowly over 7 days with a low level of urinary excretion, whereas the PEGylated conjugates ^225^Ac-Macropa-PEG_4/8_-YS5 (8.79 ± 1.09%, 8.30 ± 4.35%) demonstrated 2-fold higher kidney excretion (Figure 2F). All conjugates demonstrated a similar, lower level of fecal elimination than their urinary excretion over 7 days (Figure S24). Thus, the addition of the PEG linker increased the rate of urinary excretion.

### PET/CT Imaging of ^134^Ce-Macropa-PEG_0/4/8_-YS5 demonstrates potential theranostic applications

**Figure 3:**
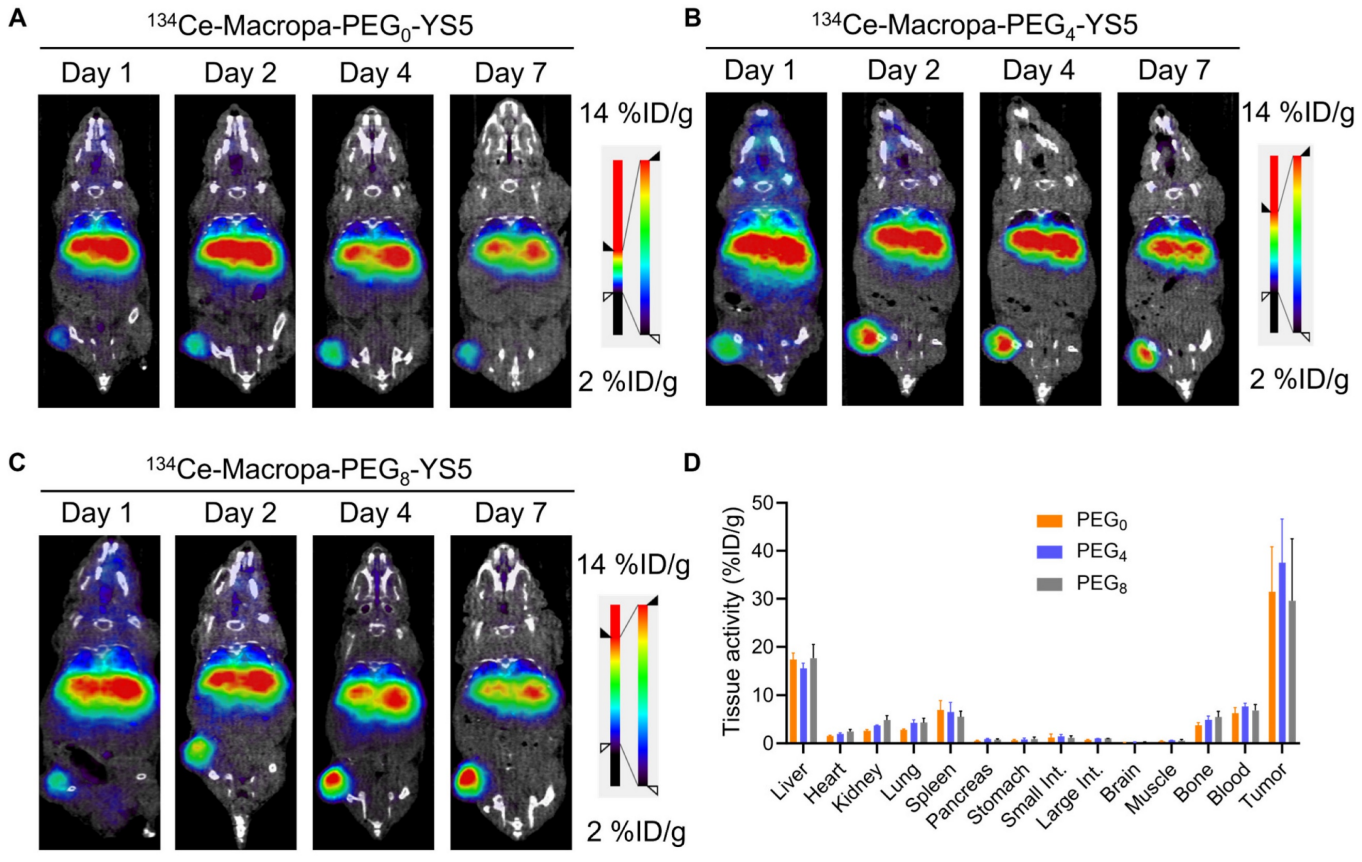
µPET/CT imaging of ^134^Ce-Macropa-YS5 agents as a theranostic pair for ^225^Ac therapy. Coronal µPET/CT images of mouse-bearing subcutaneous 22Rv1 xenografts at days 1, 2, 4, and 7 after administering the respective radio conjugates by tail vein demonstrate increased tumoral uptake over time. A) ^134^Ce-Macropa-PEG_0_-YS5. B) ^134^Ce-Macropa-PEG_4_-YS5. C) ^134^Ce-Macropa-PEG_8_-YS5. D) Ex vivo biodistribution after 7 days of PET Imaging (n = 4 mice for each group). The respective maximum intensity projection µPET/CT images are shown in Figure S25 in the supplementary appendix.

We evaluated the imaging capability of ^134^Ce-Macropa-PEG_0/4/8_-YS5 radioimmunoconjugates as PET imaging agents. Greater than 95% radiolabeling conversions were observed with corresponding high (>95%) radiochemical purity of the isolated product. PET imaging of ^134^Ce-Macropa-PEG_0/4/8_-YS5 conjugates was performed over 7 days in 22Rv1 xenograft mice. All three conjugates exhibited a gradual increase in tumor uptake over time, and maximum uptake was seen at 7 days post-injection. Most activity was cleared from non-targeted organs except for the liver (Figure 3A-C and Figure S25). Ex-vivo biodistribution was performed after the PET imaging 7 days post-injection. The ex-vivo biodistribution shows a maximum tumor uptake of 31.46 ± 9.4, 37.56 ± 9.11, and 29.58 ± 12.93 %ID/g for ^134^Ce-Macropa-PEG_0/4/8_-YS5 conjugates. Other non-targeted organs showed less than 5 %ID/g except for the liver (17.45 ± 1.29, 15.56 ± 1.13 and 17.72 ± 2.81 %ID/g), spleen (6.93 ± 1.94, 6.49 ± 2.03 and 17.72 ± 2.81 %ID/g), and blood (6.27 ± 1.16, 7.63 ± 0.72 and 6.85 ± 1.20 %ID/g) (Figure 3D). Interestingly, while the ^134^Ce-Macropa-PEG_0/8_-YS5 derivatives demonstrated similar biodistribution to the ^225^Ac counterparts, the ^225^Ac-Macropa-PEG_4_-YS5 demonstrated reduced uptake compared to ^225^Ac-Macropa-PEG_4_-YS5. This finding may be due at least in part to the therapeutic effect of the ^225^Ac compared to ^134^Ce at the later time point. Overall, these data demonstrate a potential theranostic application of these conjugates in prostate cancer models.

### In vitro analysis, autoradiography, histology, and DNA damage staining for ^225^Ac-Macropa-PEG_4_-YS5

**Figure 4:**
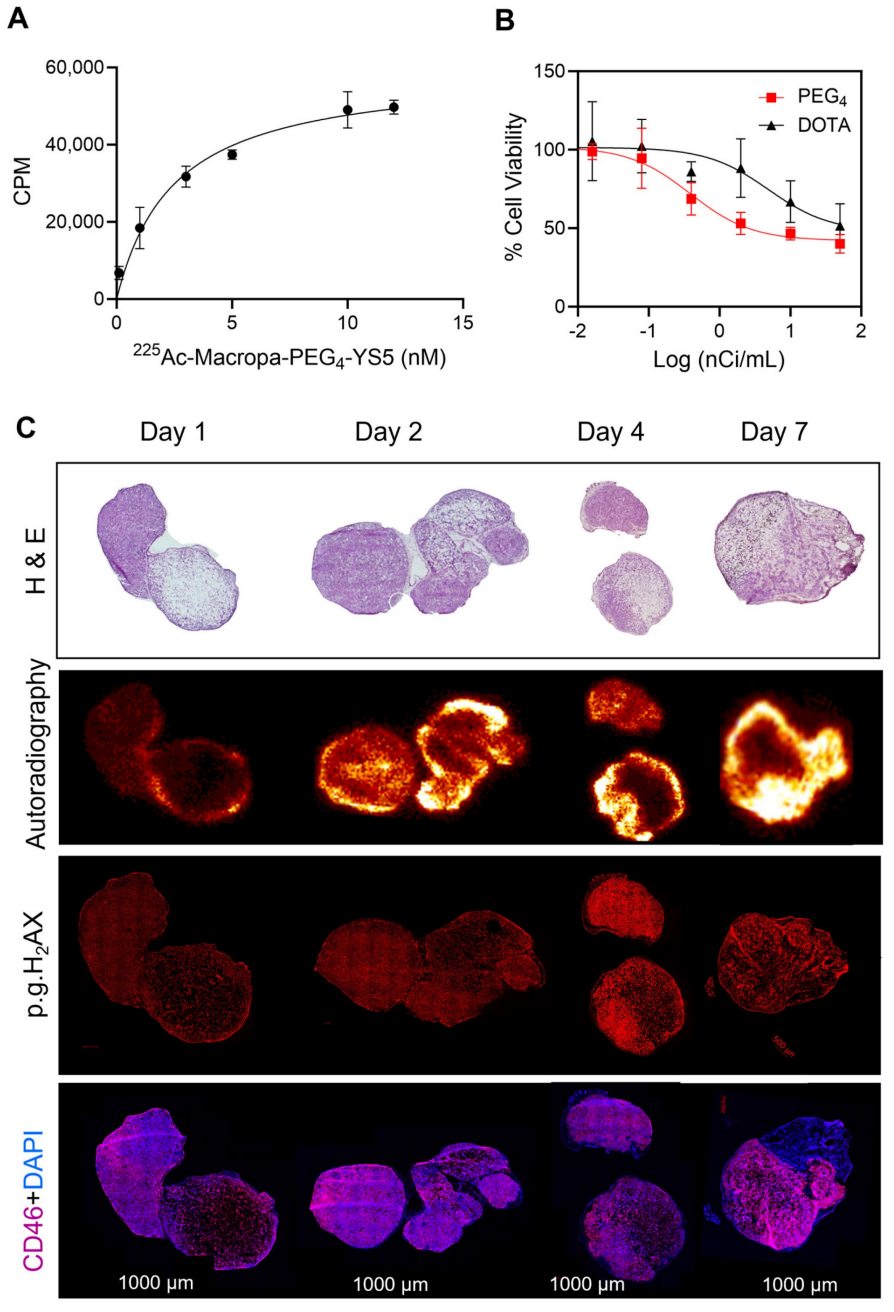
^225^Ac-Macropa-PEG_4_-YS5 shows a high binding affinity for 22Rv1 cells, efficient cell killing, and elicits a robust DNA damage response which correlates with radiopharmaceutical uptake *in vivo*. A) Saturation binding assay curve of ^225^Ac-Macropa-PEG_4_-YS5 in 22Rv1 cells. B) Activity-dependent cell death with ^225^Ac-Macropa-PEG_4_-YS5; improved cell killing seen in ^225^Ac-Macropa-PEG_4_-YS5 vs ^225^Ac-DOTA-YS5. C) Correlation of histology (H&E), immunofluorescence (phospho-γH2AX), and digital autoradiographic images after treatment with ^225^Ac-Macropa-PEG_4_-YS5 over 7 days (n = 3). Data represented as Mean ± SD (n = 3).

The properties of ^225^Ac-Macropa-PEG_4_-YS5 were evaluated for prostate cancer treatment in the 22Rv1 cell line. Previous studies in our lab have shown that 22Rv1 cells have high CD46 expression. Furthermore, a recent study with ^225^Ac-DOTA-YS5 showed excellent anti-tumor efficacy against 22Rv1 xenograft. 22Rv1 xenografts demonstrate rapid growth in athymic nude mice and are notably radioresistant, setting a high bar for in vivo effectiveness 38. ^225^Ac-Macropa-PEG_4_-YS5 with a concentration of 7 nM incubated with 22Rv1 cells showed 7.6 ± 0.3% cell binding. In contrast, it was reduced to 0.58 ± 0.03% when blocked with a 10-fold excess of cold YS5 (Figure S26A). Next, the binding affinity was assayed via saturation binding assay, and the dissociation constant of ^225^Ac-Macropa-PEG_4_-YS5 was found to be 2.5 ± 1.2 nM, compared to 25.4 ± 8.2 nM for ^225^Ac-DOTA-YS5 (Figure 4A and Figure S26B). Finally, a cell-killing assay revealed a gradual activity-dependent decrease in cell viability with a half-minimal inhibitory concentration (IC_50_) of 0.015 ± 0.21 kBq/mL for ^225^Ac-Macropa-PEG_4_-YS5. In contrast, a higher IC_50_ of 0.16 ± 0.8 kBq/mL for ^225^Ac-DOTA-YS5 was observed (Figure 4B). A non-targeted control ^225^Ac-DOTA and ^225^Ac-Macropa induced minimal toxicity at high concentrations but did not reach IC_50_ concentrations (Figure S26C) 39. These results demonstrate that the ^225^Ac-Macropa-PEG_4_-YS5 has a high binding affinity and cytotoxicity against 22Rv1 cells, and the potential for an improved therapeutic window compared against the ^225^Ac-DOTA-YS5 conjugate.

To correlate the radiopharmaceutical distribution and its effects, we administered ^225^Ac-Macropa-PEG_4_-YS5 to 22Rv1 prostate cancer xenograft mice, and digital autoradiography, with histological and phospho-γH2AX staining, was performed on tumor sections. As depicted in Figure 4C, digital autoradiography revealed a heterogeneous distribution of radioactivity on days 1, 2, 4, and 7. The non-uniform distribution corresponds to the viable regions detected in the H&E images, with absent binding in necrotic regions. The CD46 was homogenously expressed except for necrotic regions seen in H & E images. Additionally, treatment with ^225^Ac-Macropa-PEG_4_-YS5 induced DNA damage, as evidenced by the colocalization of γH2AX foci with the activity distribution.

### Chronic toxicity evaluation of ^225^Ac-Macropa-PEG_4_-YS5 in athymic nude mice

^225^Ac-Macropa-PEG_4_-YS5 was selected for treatment studies due to its high tumor uptake and favorable tumor-to-background ratios. We evaluated the chronic toxicity of 4.625 and 9.25 kBq doses for ^225^Ac-Macropa-PEG_4_-YS5 in non-tumor-bearing male nu/nu athymic mice with saline as a control group (Figure S27A). Over 125 days, the body weight and general condition were monitored. On day 57, one animal in the 4.625 kBq cohort was found dead without explaining illness or weight loss, while all other mice survived. No body weight loss was observed (Figure S27A). At day 125, the mice were euthanized, laboratory testing was conducted, and the organs were evaluated by histopathology. As anticipated, mild to moderate renal toxicity was observed in 4.625 and 9.25 kBq doses. The kidney injury consists predominantly of increased glomerular fibrin deposition, reduced capillaries in numerous glomeruli, and mild to moderate atrophy of cortical tubules (Figure S27B). In this case, mild to moderate renal toxicity was observed on histology for both doses (albeit with no detectable change on renal function blood testing). It is conceivable that a continuous redistribution of the daughter isotope Bi-213 may contribute to mild kidney toxicity. Importantly, we did not observe significant toxicity in other major organs. Liver, kidney function (Figure S28), and blood analysis results revealed no statistically significant changes in blood parameters (Figure S29).

### ^225^Ac-Macropa-PEG_4_-YS5 therapy induces prolonged survival in 22Rv1 Prostate cancer xenografts

**Figure 5:**
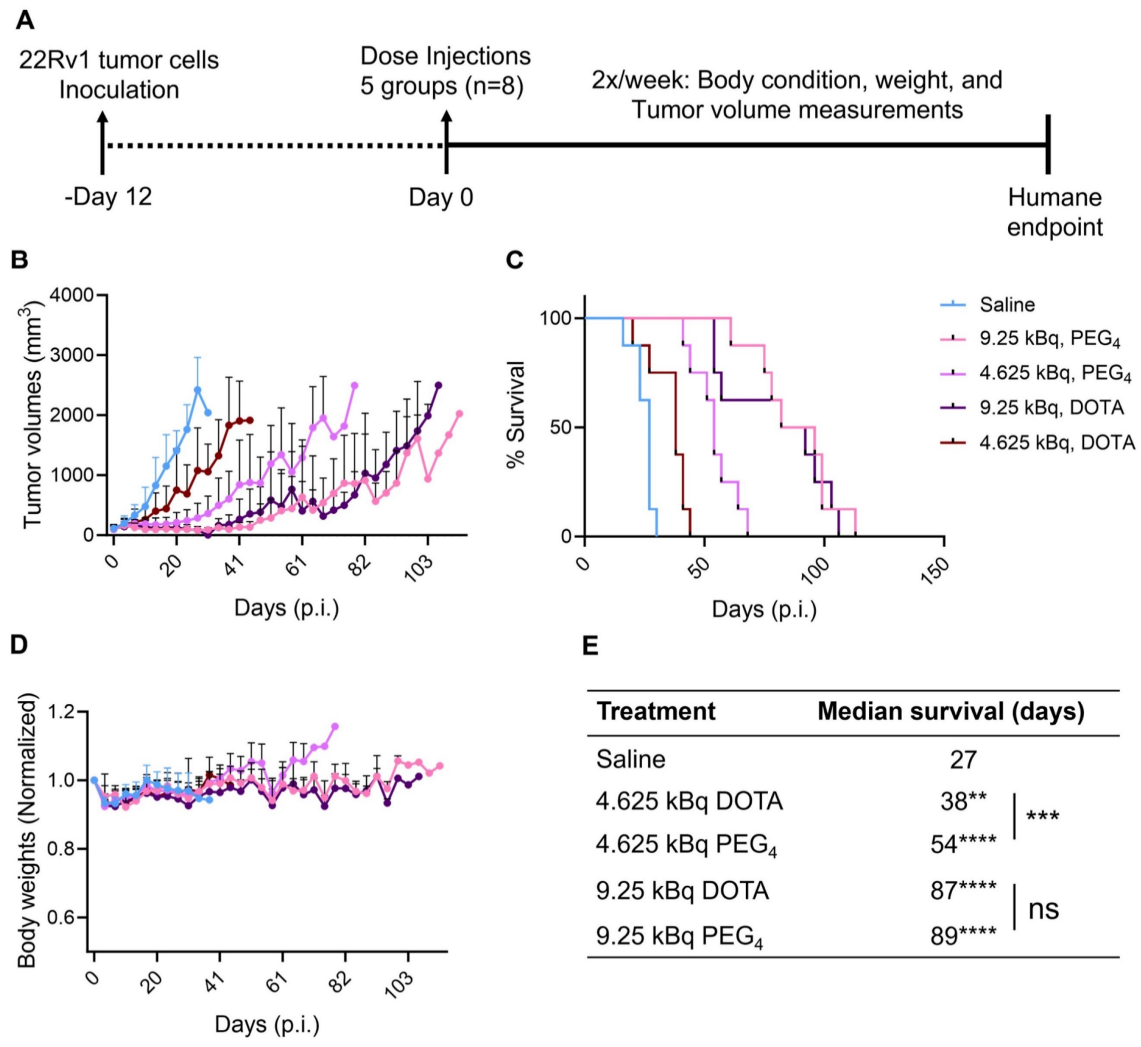
** A**) Schematic illustration of single-dose treatment for ^225^Ac-Macropa-PEG_4_-YS5 and ^225^Ac-DOTA-YS5 in subcutaneous 22Rv1 xenografts with 4.625 and 9.25 kBq doses. B) Average tumor volumes after the treatment. C) Percent survival as a function of time for the treatment and vehicle cohorts d) Normalized body weights. E) The median survival days and statistical analysis for the single treatment group (n = 8 per cohort), **p = 0.0052, ***p = 0.0005, ****p < 0.0001.

After encouraging results from the in vitro studies, the therapeutic potential for ^225^Ac-Macropa-PEG_4_-YS5 was assessed in CD46 expressing cell-derived 22Rv1 xenografts and compared with the ^225^Ac-DOTA-YS5. Mice bearing 22Rv1 subcutaneous xenografts were randomized into 5 groups (n = 8 mice per group). Animals were given a single dose, 4.625 or 9.25 kBq of ^225^Ac-Macropa-PEG_4_-YS5, ^225^Ac-DOTA-YS5, or saline vehicle by tail vein injection. Treatment with ^225^Ac-Macropa-PEG_4_-YS5 showed a dose-dependent efficacy (Figure 5A-E). Tumors in the 4.625 kBq cohort for ^225^Ac-DOTA-YS5 grew rapidly at a similar rate to the vehicle saline cohort and reached the endpoint with a median survival of 38 days compared to 27 days for the saline cohort. In contrast, ^225^Ac-Macropa-PEG_4_-YS5 showed a prolonged, significant tumor growth inhibition with a median survival of 54 days (p = 0.0005). The higher activity dose 9.25 kBq cohorts for both radioimmunoconjugates also showed a prolonged tumor growth inhibition. The tumor growth inhibition started approximately 9-16 days post-injection; however, the tumor regrowth began after 41 days. The overall median survival for ^225^Ac-Macropa-PEG_4_-YS5 was 89 days, and ^225^Ac-DOTA-YS5 was 87 days, significantly higher than the vehicle group (p<0.0001); however, no significant differences were found between the radio-immunoconjugates at this higher dose level. The reasons for the discrepant behavior at the two different activity levels are unclear, but we speculate that higher activity effectively treats tumors regardless of their tumor uptake. In contrast, lower administered activities require a high tumor uptake to elicit such a significant radiation dose and, consequently, antitumor response. A slight decrease in body weight was observed immediately post-injection in both treatment and control groups, which resolved by day 16. No other signs of toxicity were noted for all the cohorts.

### Fractionated therapy regimen demonstrates greatly improved therapeutic efficacy for ^225^Ac-Macropa-PEG_4_-YS5

**Figure 6:**
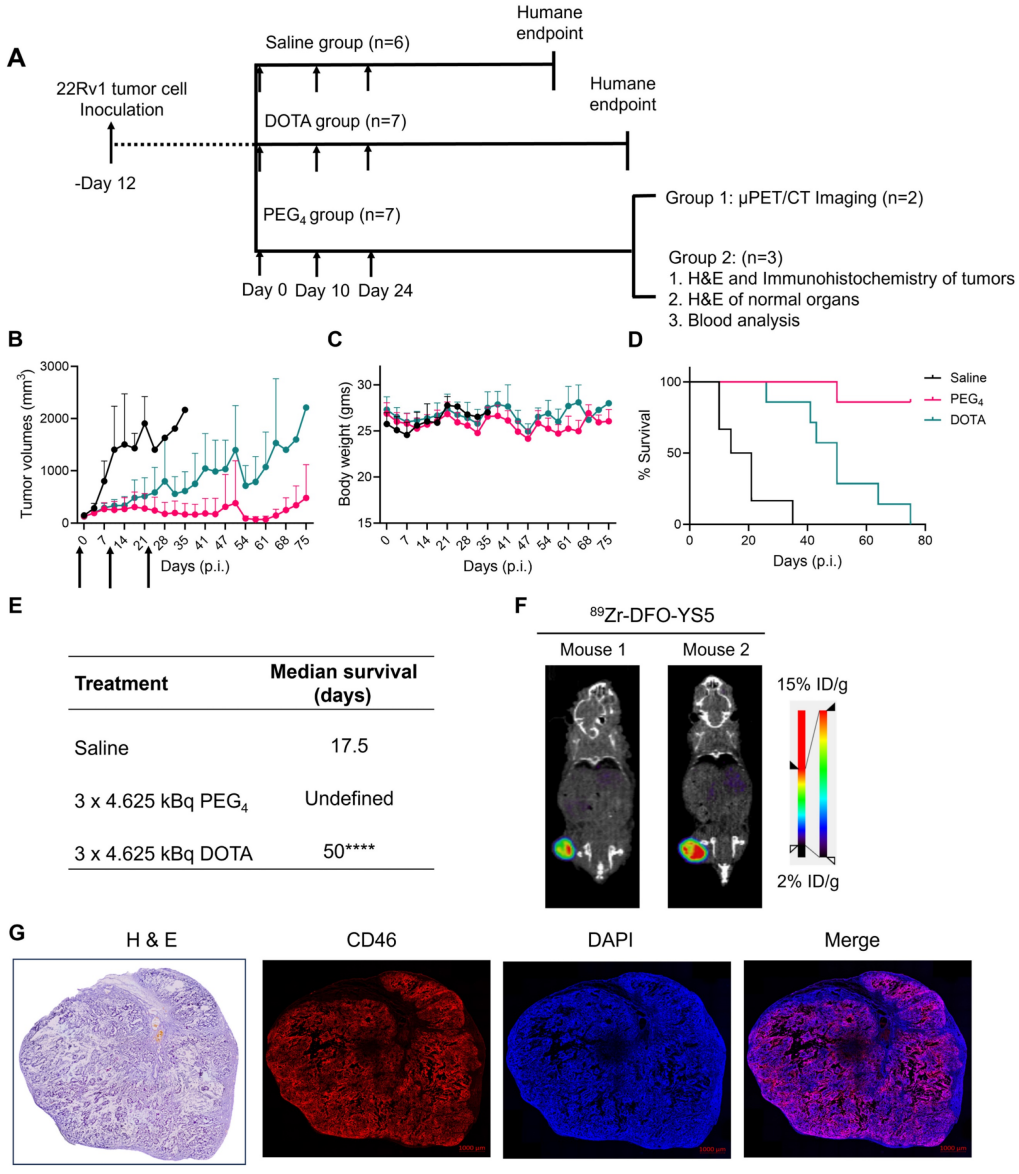
^225^Ac-Macropa-PEG_4_-YS5 causes improved therapeutic outcomes compared against ^225^Ac-DOTA-YS5 in a fractionated dosing study. A) Schematic illustration for fractionated treatment study of ^225^Ac-Macropa-PEG_4_-YS5 and ^225^Ac-DOTA-YS5 with a 3 x 4.625 kBq dose in 22Rv1 xenografts. B) Average tumor volumes, C) body weight, and D) Percent survival as a function of time for the treatment and vehicle cohorts. E) The median survival days and statistical analysis for the fractionation treatment group (n = 6 for saline group, n = 7 for ^225^Ac-Macropa-PEG_4_-YS5 and ^225^Ac-DOTA-YS5 group. F) Micro PET/CT imaging using ^89^Zr-DFO-YS5 in the group previously treated with ^225^Ac-Macropa-PEG_4_-YS5 (3 x 4.625 kBq) in 22Rv1 xenografts. Coronal micro-PET/CT images were obtained on day 4 post-injection (n = 2, group 1) after the end of the study. G) Histology and immunofluorescence imaging of group 2 mice showing persistent CD46 expression in tumors following 3 x 4.625 kBq administrations of ^225^Ac-Macropa-PEG_4_-YS5 (n = 3, group 2). ****p < 0.0001, undefined: not reached endpoint.

A fractionated therapy regimen was also tested to compare the efficacy of ^225^Ac-Macropa-PEG_4_-YS5 and ^225^Ac-DOTA-YS5. The mice were randomized into 3 groups (n = 7 for treatment cohorts and 5 mice for saline vehicle). Three fractionated doses of 4.625 kBq of the radiopharmaceutical, or vehicle control, were administered intravenously to the mice bearing 22Rv1 xenografts on day 0, day 10, and day 24 (Figure 6A). As shown in Figure 6B-E, the 3 x 4.625 kBq doses of ^225^Ac-Macropa-PEG_4_-YS5 delayed tumor growth significantly compared to the ^225^Ac-DOTA-YS5 and saline control group. The median survival for ^225^Ac-DOTA-YS5 was 50 days, while 17.5 days for the vehicle cohort was noted. The endpoint was not reached at 75 days for the ^225^Ac-Macropa-PEG_4_-YS5 group, and the remaining mice underwent correlative studies as noted below. These data demonstrate that the ^225^Ac-Macropa-PEG_4_-YS5 conjugate showed improved therapeutic efficacy over ^225^Ac-DOTA-YS5 in fractionated and single-dose therapy regimens.

### Correlative studies in mice receiving fractionated doses of ^225^Ac-Macropa-PEG_4_-YS5

On day 75, five out of seven animals in the ^225^Ac-Macropa-PEG_4_-YS5 cohort were still alive with detectable tumors ranging from 0.27 - 1.7 cc. Animals were divided into two groups. One group of n = 2 underwent ImmunoPET Imaging with ^89^Zr-DFO-YS5. Both mice demonstrated uptake in the tumor (Figure 6F and Figure S30). The other group of n = 3 was euthanized, and blood and chemistry studies were performed, with findings similar to the chronic toxicity study. Histologic analysis was also similar, with the exception of one mouse, which showed centrizonal inflammation and focal centrizonal necrosis in the liver, perivascular and peribronchiolar fibrosis and inflammation in the lungs, with focal acute inflammation, and scattered histiocytic aggregates in the spleen (Figures S31, 32). Subsequently, CD46 expression changes within the tumor were assayed following radiopharmaceutical treatment. As illustrated in Figure 6G, CD46 exhibited uniform expression patterns, except for specific regions of central necrosis that were evident in the H&E stain. Moreover, nuclear staining with DAPI revealed colocalization with CD46 expression. Correlative studies in the surviving mice after ^225^Ac-Macropa-PEG_4_-YS5 demonstrated preserved CD46 expression in the mice bearing tumors as visualized by both ImmunoPET and immunohistochemistry. Taken together, these studies suggest promise for a fractionated dosing scheme at the time of clinical translation of this methodology.

## Discussion

Targeted Alpha Particle Therapy (TAT) represents a promising approach for delivering radiation selectively to tumors, maximizing therapeutic efficacy while minimizing toxicity. Among the other alpha emitters, Ac-225 has stood out due to its potency, availability, and promising clinical results 2,40. Clinical responses to radiopharmaceuticals like ^225^Ac-DOTATATE 14 and ^225^Ac-PSMA-617 7 have been observed, including in patient's refractory to ^177^Lu-based treatments. Accordingly, we previously developed the CD46 targeted, ^225^Ac labeled antibody, ^225^Ac-DOTA-YS5, and demonstrated its efficacy in preclinical models of prostate cancer. An advantage of this technology is that it has efficacy in a wide range of preclinical models, including PSMA-negative tumors, both neuroendocrine prostate cancer and adenocarcinoma. In this study, we built on this foundation and developed the short PEGylated radioimmunoconjugate ^225^Ac-Macropa-PEG_4_-YS5, which has exhibited superior biodistribution and treatment results in prostate cancer xenografts.

Given Ac-225's longer half-life, it is well-suited for antibody-based TAT applications. Traditionally, DOTA has been the gold standard chelator for Ac-225, but its reaction kinetics with Ac^3+^ at room temperature and in vivo stability have posed challenges. The macropa chelator, developed by Thiele *et al.*, has emerged as a promising alternative 32. Its advantages include room temperature radiolabeling and thermodynamically stable radiocomplexes, making it preferable for our study. We modified the macropa chelator by introducing short PEG_4/8_ linkers to generate lead compounds, Macropa-PEG_4/8_-TFP esters 33, which were conjugated with the YS5 antibody. Remarkably, we achieved >95% radiochemical yields for all conjugates, regardless of the number of chelators per antibody. Conversely, DOTA-YS5 failed to exhibit such conversions, even at high chelator-to-antibody ratios (Figure 1). Interestingly, while macropa NCS-based conjugates have previously demonstrated high stability 41,42, our study revealed that ^225^Ac-Macropa-PEG_0_-YS5 displayed significant instability, likely due to the thioamide linkage between Macropa and YS5. In contrast,^ 225^Ac-Macropa-PEG_4/8_-YS5 exhibited high in vitro stability, likely attributed to the amide linkage 37.

Recent studies have highlighted the benefits of inserting short PEG linkers in radioimmunoconjugates, such as improved tumor-to-background ratios and faster clearance 31. Guillou *et al.* reported that PEGylation of a radiolabeled antibody led to different metabolic pathways, reducing nonspecific accumulation and retention in background organs 43. Our biodistribution studies echoed these findings, showing higher tumor uptake and reduced off-target binding for PEGylated ^225^Ac-Macropa-PEG_4/8_-YS5 compared to non-PEGylated ^225^Ac-Macropa-PEG_0_-YS5 and DOTA-YS5 (Figure 2). Notably, higher chelator-to-antibody ratios also resulted in increased liver uptake, indicating a potential immunoreactivity loss due to extensive antibody modification (Table S4,5) 44. Furthermore, the prior literature with Macropa NCS-based conjugates demonstrated high liver uptake, which might be due to the instability of the thioamide linkage 41,42. Similar observations were found with ^225^Ac-Macrop-PEG_0_-YS5 conjugate as such observations were not found for ^225^Ac-Macropa-PEG_4/8_-YS5 conjugates including ^225^Ac-DOTA-YS5 conjugate and other non-NCS based Macropa conjugates 37. Moreover, the metabolic analysis demonstrated that PEGylated derivatives underwent faster renal clearance, reinforcing their suitability for therapeutic applications.

The concept of theranostic-matched imaging pairs has gained traction due to their similar pharmacokinetics with therapeutic isotopes 45-47. We and others have proposed ^134^Ce/La as an imaging surrogate for ^225^Ac-based radioligand therapies. Our previous communication confirms that ^134^Ce-Macropa complex is highly stable in saline and human serum buffers until 7 days. We also observed that the ^134^La ejection from the ^134^Ce/La-conjugates was due to the nuclear recoil effect (chromatographically) although the ^Nat^La-MACROPA (K_LnL_=14.91) and ^Nat^Ce- MACROPA (K_LnL_=15.1) are highly stable. The in vivo re-chelation of ^134^La may be less likely, and in vivo redistribution of^ 134^La is evident 33. The PET Imaging and biodistribution analysis exhibited high tumor uptake for ^134^Ce-Macropa-PEG_0/4/8_-YS5 conjugates (Figure 3). This offers the potential for Macropa-PEG conjugates to serve both therapeutic and imaging purposes, mitigating chelator-specific differences in vivo pharmacokinetics.

^225^Ac-Macropa-PEG_4_-YS5 has been selected for treatment studies due to its notable tumor uptake and favorable tumor-to-background ratios. In a prior investigation, we assessed the acute and chronic toxicity of 4.625, 9.25, and 18.5 kBq doses of ^225^Ac-DOTA-YS5. Remarkably, no acute toxicity was observed across any of these doses. However, the chronic toxicity assessment at the 18.5 kBq dose level revealed severe kidney damage 25. Given these findings, we evaluated the chronic toxicity of 4.625, and 9.25 kBq doses for ^225^Ac-Macropa-PEG_4_-YS5. In this case, mild to moderate renal toxicity was observed on histology for both doses (albeit with no detectable change on renal function blood testing), a phenomenon not observed with ^225^Ac-DOTA-YS5. We attribute this discrepancy to two potential factors: firstly, the long-term in vivo stability of the ^225^Ac-Macropa-PEG_4_-YS5 complex, and secondly, its superior tumor uptake when compared to ^225^Ac-DOTA-YS5. It is conceivable that a continuous redistribution of the daughter isotope Bi-213 may contribute to mild kidney toxicity in both scenarios. Importantly, we did not observe significant toxicity in other major organs.

We investigated the therapeutic efficacy of ^225^Ac-Macropa-PEG_4_-YS5 in a mildly PSMA-positive prostate cancer model (22Rv1). High doses of 4.625, and 9.25 kBq exhibited a prolonged survival rate comparable to that of the ^225^Ac-DOTA-YS5 conjugate. No significant differences in survival rates were observed between these two conjugates. However, the low dose of 4.625 kBq displayed notable distinctions in terms of antitumor response and overall survival compared to ^225^Ac-DOTA-YS5. In fact, the overall survival rates for ^225^Ac-DOTA-YS5 resembled those of the control group. We hypothesize that higher doses effectively eradicate tumors regardless of their tumor uptake, while lower doses require a high tumor uptake to elicit such a significant radiation dose and consequently antitumor response. This was observed specifically for the PEGylated ^225^Ac-Macropa-PEG_4_-YS5 in contrast to the non-PEGylated ^225^Ac-DOTA-YS5 (Figure 5). These results were further explored in a fractionated dosing study, where a marked improvement was again seen comparing ^225^Ac-Macropa-PEG_4_-YS5 to ^225^Ac-DOTA-YS5. Correlative studies in the surviving mice after ^225^Ac-Macropa-PEG_4_-YS5 demonstrated preserved CD46 expression in the mice bearing tumors as visualized by both immunoPET and immunohistochemistry (Figure 6). Taken together, these studies suggest promise for a fractionated dosing scheme at the time of clinical translation of this methodology.

Like many others, our previous study highlights the kidney as a major dose-limiting organ during Ac-225 radioligand therapy 25. Due to the recoil effect, the redistribution of the daughter radioisotope Bi-213 from the Ac-225 decay chain accumulates in the kidneys post-chelation. Nephrotoxicity has also been observed in other antibody-based radioligand therapies, given that metabolites clear through renal pathways. This observation may also be seen targeting vectors such as antibody fragments, antibody mimetics, peptidomimetics, and small molecules 48. In response to these challenges, emerging strategies aim to mitigate radiation-induced nephrotoxicity. These include using cleavable linkers 49, pre-targeting methods 50, and chelation therapy. Moreover, short PEG linkers have enhanced tumor-to-target background ratios 51. While higher doses have demonstrated excellent antitumor responses but exhibited high renal toxicity. Our study strongly supports the use of short PEG linkers along with high affinity chelators, to improve tumoral radiation delivery as well as therapeutic efficacy.

## Conclusion

Our study introduced ^225^Ac-Macropa-PEG_4_-YS5, an innovative radioimmunoconjugate utilizing optimized chelation chemistry and improved linker technology linked to a specific cancer-specific antibody with low background tissue binding. Compared to the previously described ^225^Ac-DOTA-YS5, it demonstrated remarkable stability, improved tumor-to-background ratios, and treatment efficacy in the prostate cancer 22Rv1 xenografts. While our study identified mild to moderate renal toxicity at the highest dose levels, it also highlighted the potential for significant antitumor responses, emphasizing the importance of optimizing short PEG linkers for a balanced therapeutic efficacy and nephrotoxicity profile. Additionally, we demonstrated the feasibility of theranostic matched pair imaging, using ^134^Ce/La for imaging alongside ^225^Ac. Ongoing research efforts aim to mitigate toxicities and maximize TAT's clinical utility, underscoring the promising future of this approach in cancer therapy.

## Supplementary Material

Supplementary information, figures, and tables.

## Abbreviations

TAT: Targeted alpha therapy
LET: linear energy transfer
PSMA: prostate-specific membrane antigen
mCRPC: metastatic castration-resistant prostate cancer
RCY: radiochemical yields
HAS: human serum albumin
SEC: size exclusion chromatography
%ID/g: percent injected dose per gram
IC50: half-minimal inhibitory concentration
H&E: Hematoxylin and Eosin
PEG_0_: ^225^Ac-Macropa-PEG_0_-YS5
PEG_4_: ^225^Ac-Macropa-PEG_4_-YS5
PEG_8_: ^225^Ac-Macropa-PEG_8_-YS5
DOTA: ^225^Ac-DOTA-YS5
