# Supplementary material for: Development of CD46 targeted alpha theranostics in prostate cancer using 134Ce/225Ac-Macropa-PEG4-YS5
Source: Theranostics. 2024 Jan 27;14(4):1344–60. doi: 10.7150/thno.92742 (PMC10879874; doi:10.7150/thno.92742)
Supplement: Supplementary file 1 — Supplementary information, figures, and tables. [file thnov14p1344s1.pdf]

Supplemental Material

SUPPLEMENTAL METHODS

Materials

The chemicals and solvents utilized in the study were sourced from commercial suppliers and were not subjected to any purification unless specifically mentioned. YS5 antibody was prepared in accordance with a previously established method(1). TFP-PEG$_8$-TFP (with a purity greater than 95%, Catalog ID:11056) was procured from Biopharma PEG in Watertown, MA, USA. The bifunctional chelator Macropa-NCS was synthesized according to the previous procedure(*2*). Ammonium acetate (NH_4OAc) and Ethylenediaminetetraacetic acid disodium salt dihydrate (EDTA) were purchased from Sigma Aldrich located in St. Louis, MO, and were of ACS reagent grade. The stability studies utilized human serum obtained from Sigma Aldrich, and the sterile 0.9% saline was procured from APP Pharmaceuticals. VWR provided the Matrigel matrix and sterile phosphate-buffered saline (PBS). The deionized water utilized in the study had a resistivity of ≥18 MΩ cm and was obtained via the Evoqua water purification system. The radio TLC analysis utilized instant thin-layer chromatography paper impregnated with silica gel (iTLC-SG) sourced from Agilent Technologies based in Mississauga, ON, Canada.

Analytical methods

^{1}H&^{13}C-NMR were recorded on a Bruker AV III HD 400 MHz spectrometer using TMS as an internal standard (0 ppm). Mass analysis was performed on a Finnigan LTQ FT-ICR mass spectrometer equipped with an electrospray ionization source (LTQ FT, Thermo Fisher Scientific, Waltham, MA) in either positive or negative ion mode at Chemistry Mass Spectrometry Facility, B207 Stanley Hall, QB3 Institute, University of California, Berkeley, CA 94720-3220.

The mass spectrometry core facility at the University of Alberta in Edmonton, Canada, performed the Matrix-assisted laser desorption/ionization-time of flight (MALDI-TOF) analysis. For radio-TLC analysis, the Bioscan System AR-2000 Imaging Scanner equipped with winscan software from Bioscan Inc. in California, USA, was used.

Size-Exclusion Chromatography (SEC)

Size-Exclusion Chromatography (SEC) was carried out using a Merck Hitachi LaChrom Elite system with a Carroll and Ramsey associates' model 105S radioactivity detector using a BioSep 5 µm SEC-s3000 290 Å column from Phenomenex, Inc. in Torrance, CA, USA and 1X PBS buffer as a mobile phase. The flow rate for SEC was maintained at 1 mL per minute. ^{225}Ac-Macropa-

PEG$_{0/4/8}$-YS5 (~111-185 µCi was diluted in 50 µL of saline, ~45 µL) was injected into SEC HPLC and mL fractions were collected until 30 min. The collected fractions were counted on the Hidex gamma counter after 24 h secular equilibrium was reached and then plotted time versus counts per minute (cpm)

CD46 magnetic beads target binding fraction assay

Target binding fraction assay was performed with our previously described method(3, 4)

PET Imaging and ex-vivo biodistribution for ^{134}Ce-Macropa-PEG$_{0/4/8}$-YS5 conjugates

PET Imaging was performed according to the previously reported method(3).

Radiolabeling of ^{225}Ac-DOTA and Macropa

Radiolabeling was performed by following the reported protocol (3).

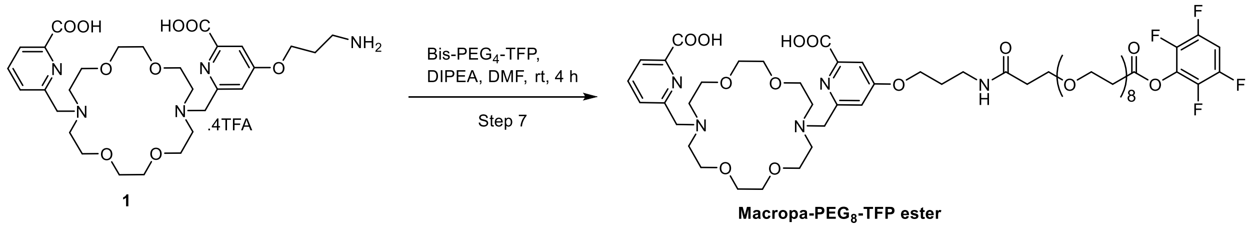

Reaction scheme 1: Synthesis of the bifunctional chelator Macropa-PEG$_8$-TFP ester. Intermediate **1** was synthesized as previously reported (3).

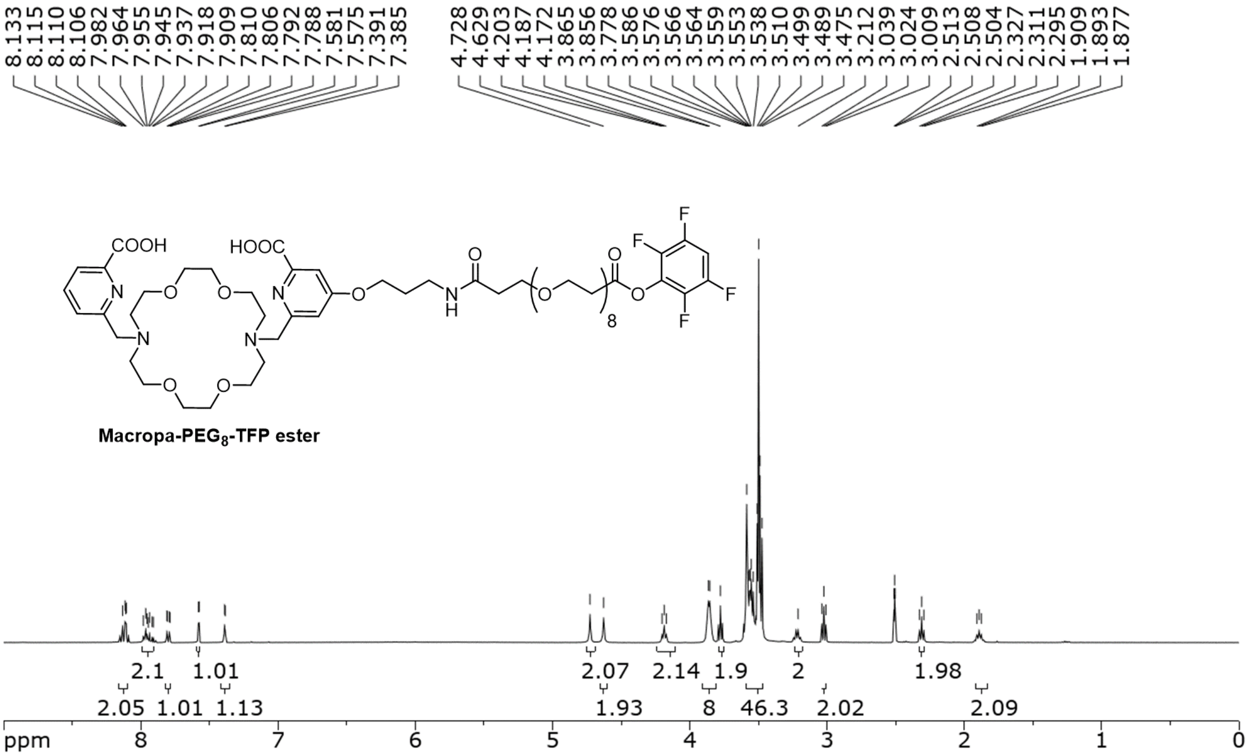

Figure S1.^{1}H-NMR of Macropa-PEG$_8$-TFP ester in DMSO-d$_6$

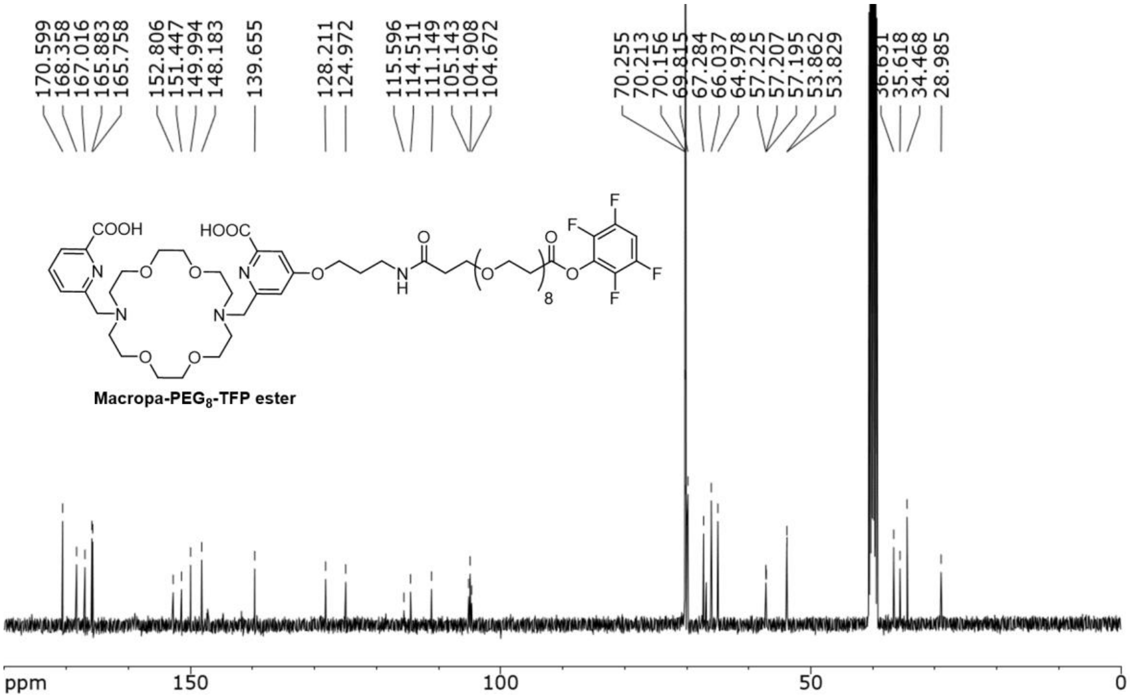

Figure S2. ^{13}C-NMR of Macropa-PEG$_8$-TFP ester in DMSO-d$_6$

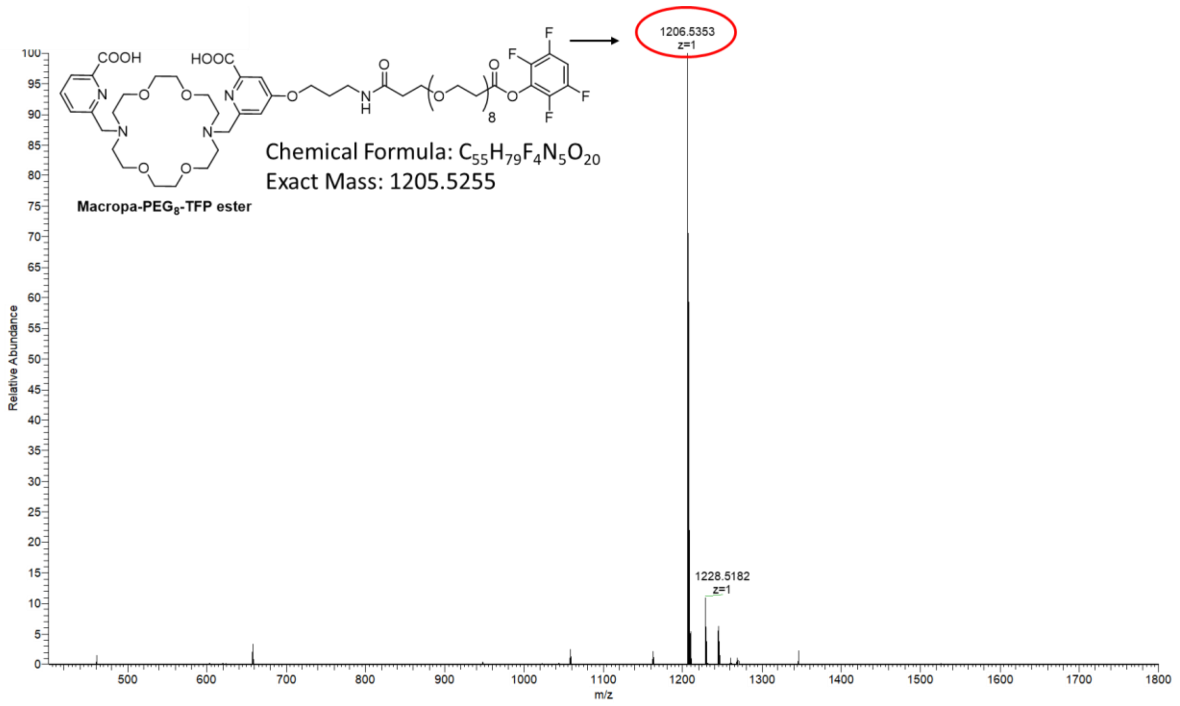

Figure S3. HRMS of Macropa-PEG$_8$-TFP ester

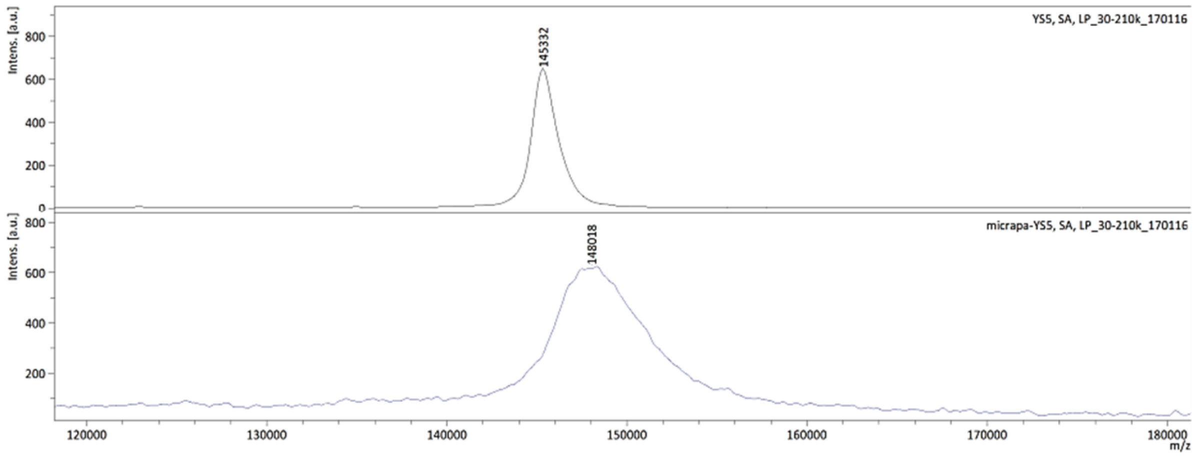

FIGURE S4. MALDI-ToF mass analysis; YS5, and Macropa-PEG$_0$-YS5 chromatograms (20 eq. of Macropa-NCS). Analysis confirms ~4.5 chelators per antibody. The mass difference between YS5 and Macropa-PEG$_0$-YS5 was divided by the mass of the Macropa-NCS compound to yield the number of chelators per Antibody YS5. Similar calculations were followed for PEG$_4$ and PEG$_8$ conjugates.

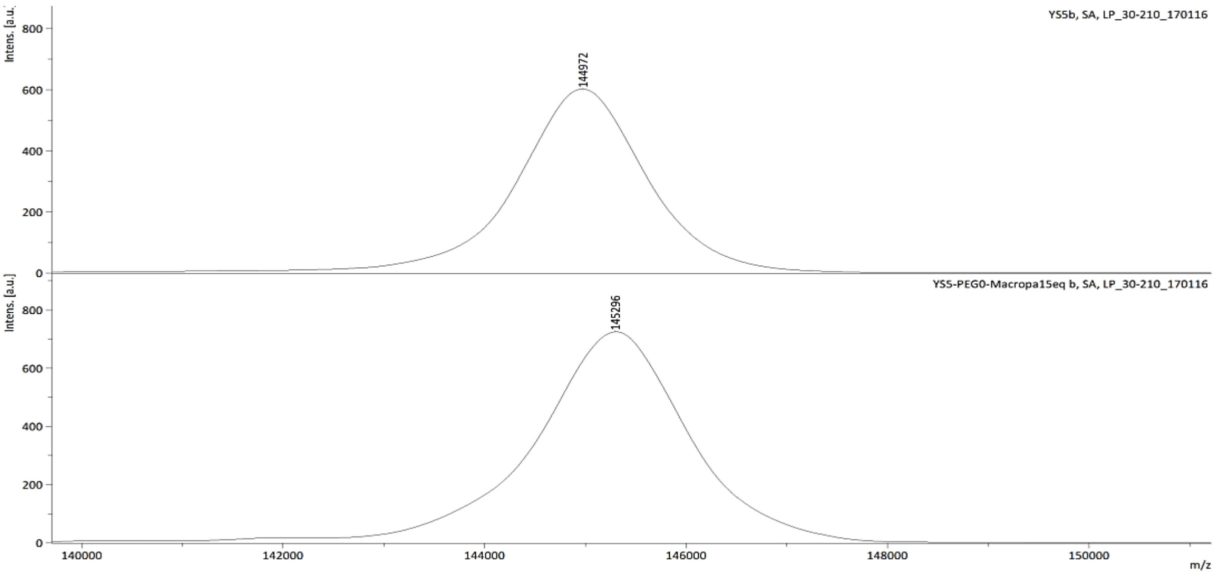

FIGURE S5. MALDI-ToF mass analysis, YS5 and Macropa-PEG$_0$-YS5 chromatograms (15 eq. of Macropa-NCS). Analysis confirms ~0.56 chelators per antibody.

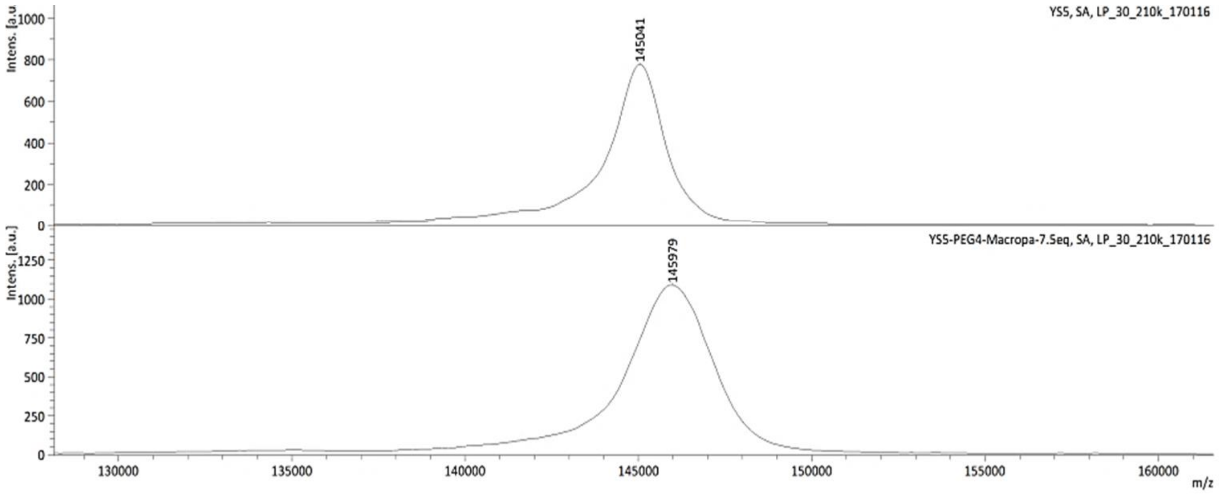

FIGURE S6. MALDI-ToF mass analysis, YS5 and Macropa-PEG$_4$-YS5 chromatograms (7.5 eq. of Macropa-PEG$_4$-TFP ester). Analysis confirms ~0.91 chelators per antibody.

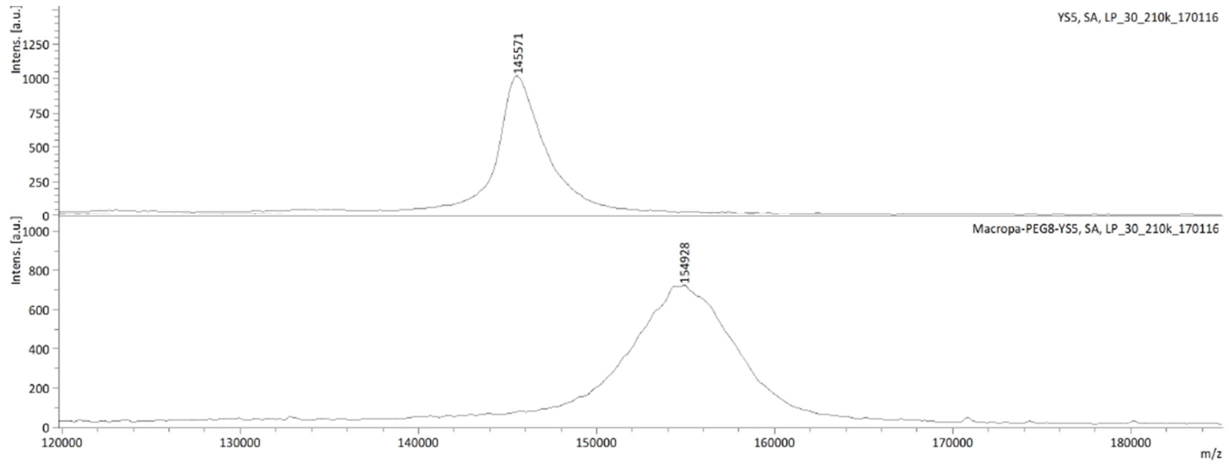

FIGURE S7. MALDI-ToF mass analysis, YS5, and Macropa-PEG$_8$-YS5 chromatograms (15 eq. of Macropa-PEG$_8$-TFP ester). Analysis confirms ~7.7 chelators per antibody.

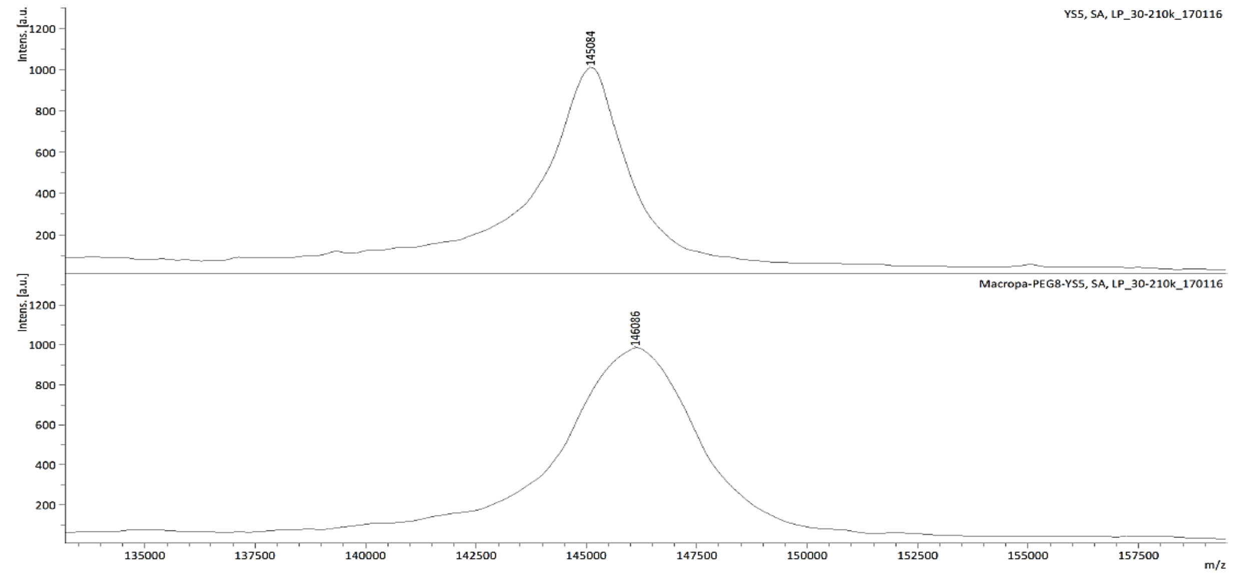

FIGURE S8. MALDI-ToF mass analysis, YS5, and Macropa-PEG$_8$-YS5 chromatograms (7.5 eq. of Macropa-PEG$_8$-TFP ester). Analysis confirms ~0.96 chelators per antibody.

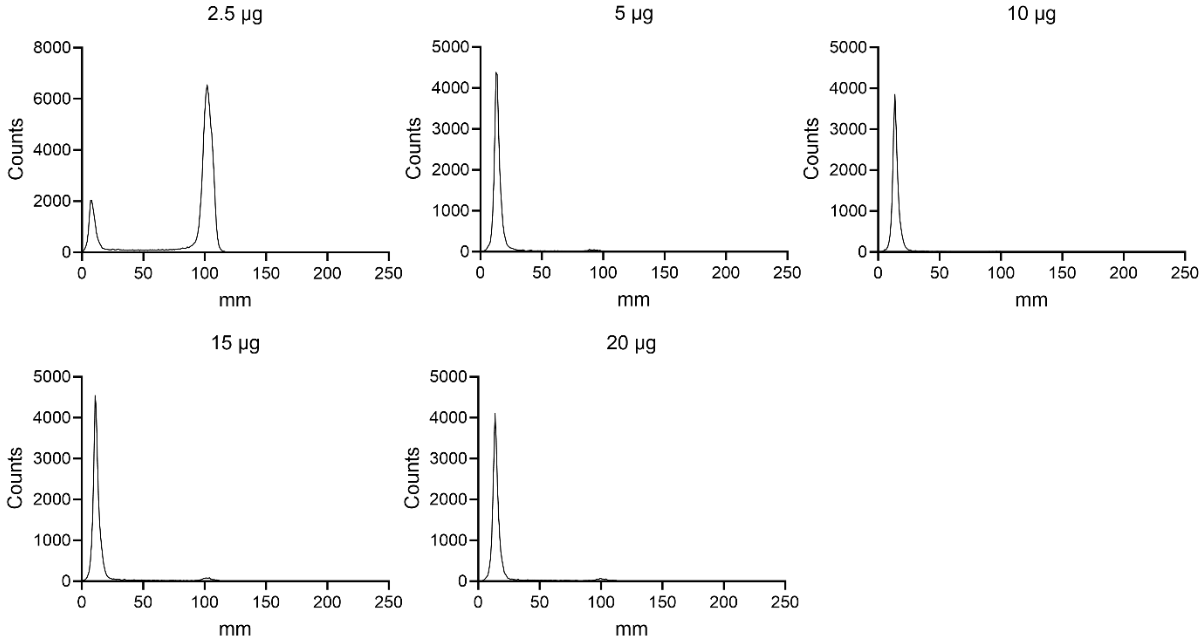

FIGURE S9. Radiolabeling of various ratios amounts of Macropa-PEG$_0$-YS5 (2.5, 5, 10, 15 and 20 µg) with Ac-225 (5 µCi) was performed and monitored by radio-iTLC-SG using 10 mM EDTA pH=5.5 as an eluent. Radio-iTLC-SG chromatograms for ^{225}Ac-Macropa-PEG$_0$-YS5 were presented; All radio-iTLC's were scanned after 30 min incubation at 30.0°C.

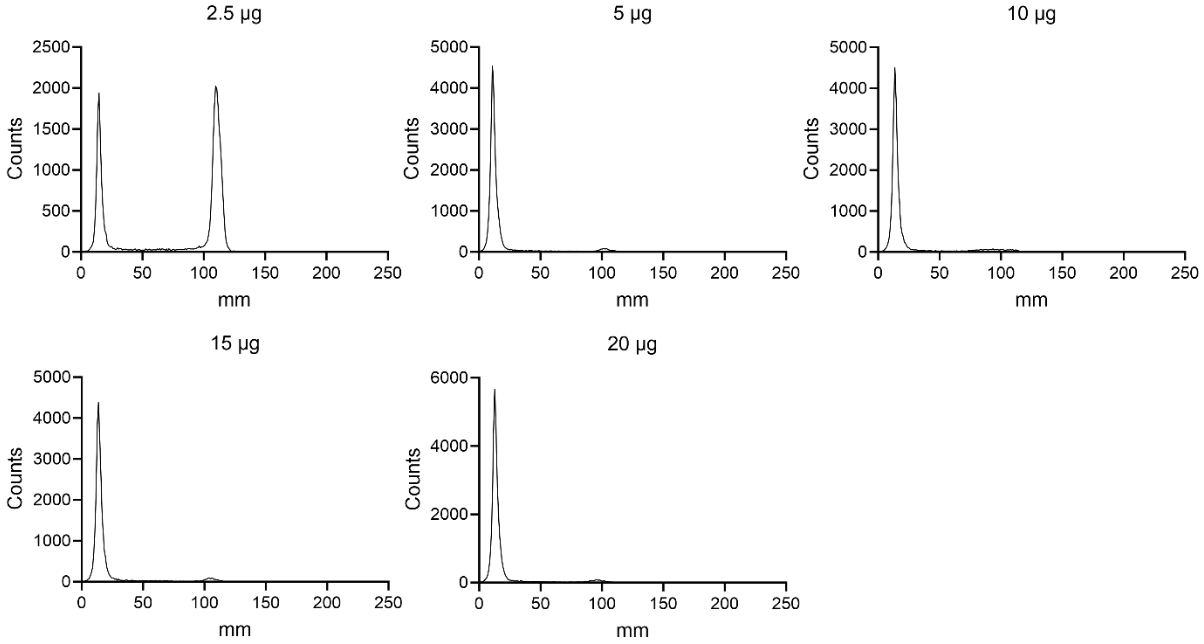

FIGURE S10. Radiolabeling of various ratios amounts of Macropa-PEG$_4$-YS5 (2.5 and 20 µg) with Ac-225 (5 µCi) was performed and monitored by radio-iTLC-SG using 10 mM EDTA pH=5.5 as an eluent. Radio-iTLC-SG chromatograms for ^{225}Ac-Macropa-PEG$_4$-YS5; All radio-iTLC's were scanned after 30 min incubation at 30.0°C.

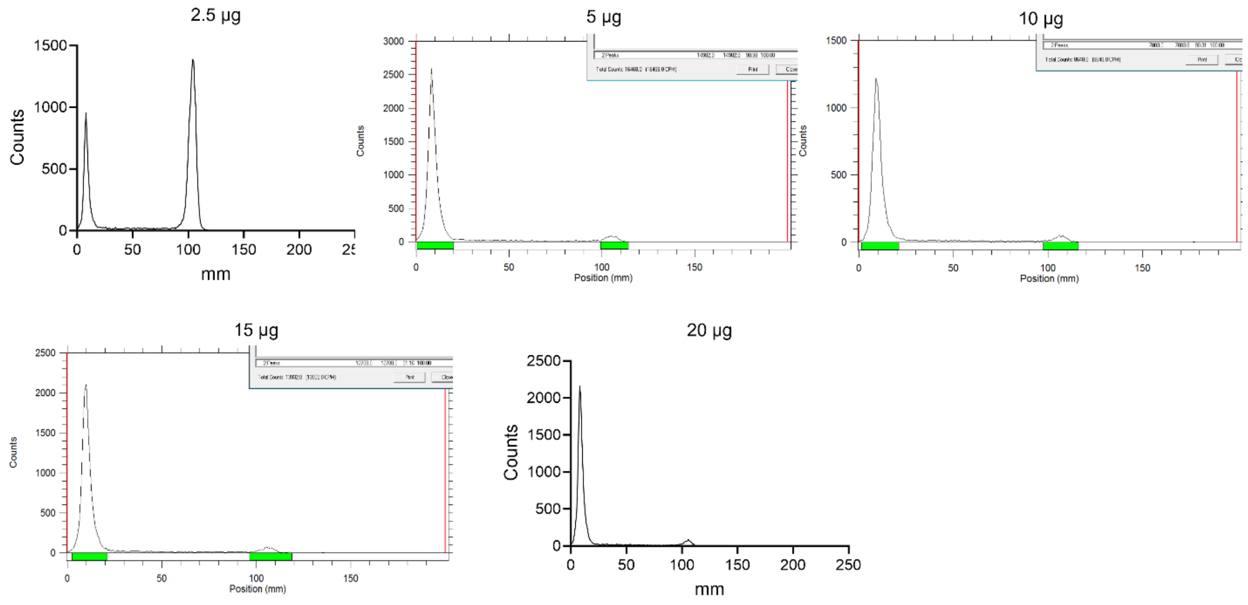

FIGURE S11. Radiolabeling of various ratios amounts of Macropa-PEG$_8$-YS5 (2.5, 5, 10, 15 and 20 µg) with Ac-225 (5 µCi) was performed and monitored by radio-iTLC-SG using 10 mM EDTA pH=5.5 as an eluent. Radio-iTLC-SG chromatograms for ^{225}Ac-Macropa-PEG$_8$-YS5; All radio-iTLC's were scanned after 30 min incubation at 30.0°C.

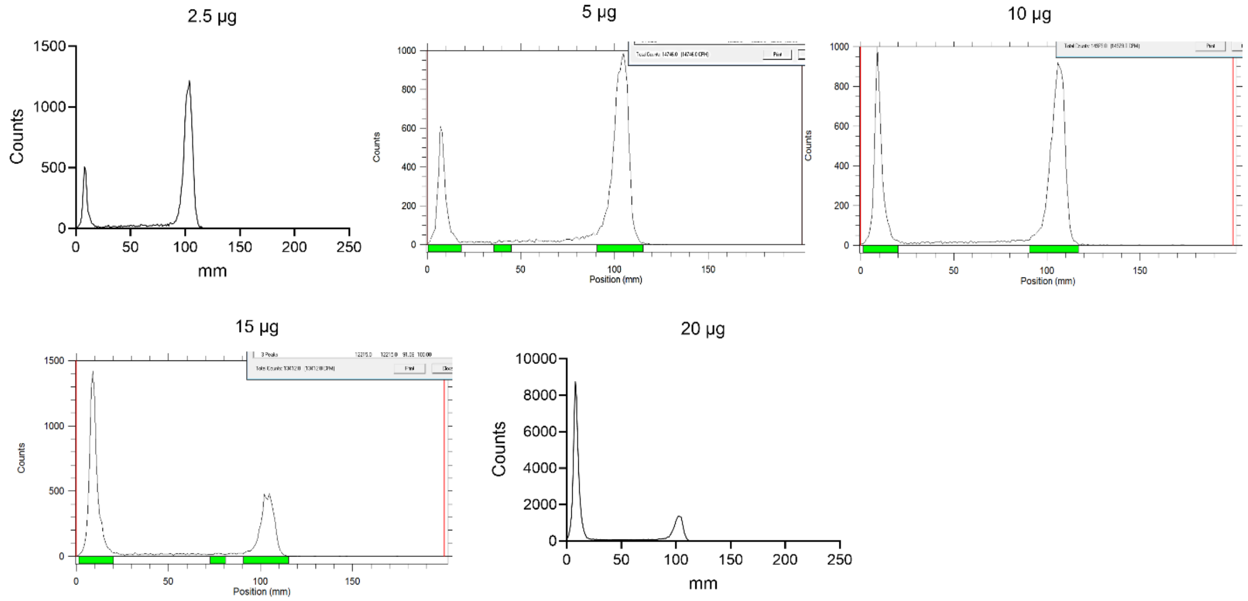

FIGURE S12. Radiolabeling of various ratios amounts of DOTA-YS5 (2.5 and 20 µg) with Ac-225 (5 µCi) was performed and monitored by radio-iTLC-SG using 10 mM EDTA pH=5.5 as an eluent. Radio-iTLC-SG chromatograms for ^{225}Ac-Macropa-PEG$_8$-YS5; All radio-iTLC's were scanned after 30 min incubation at 30.0°C.

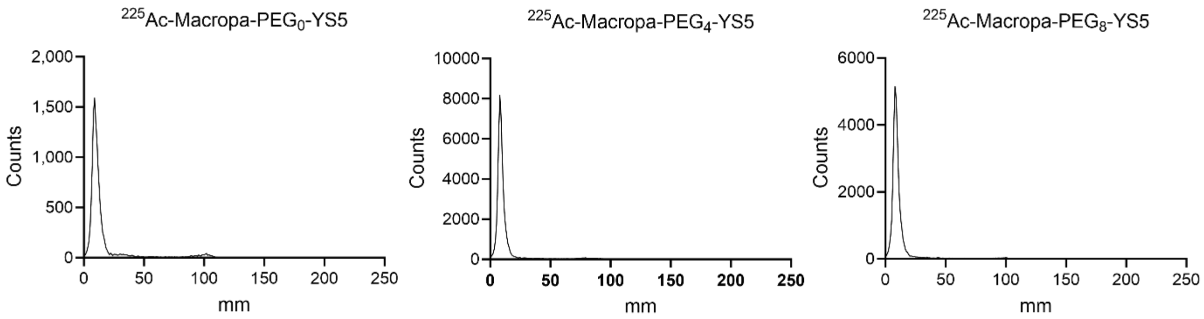

FIGURE S13. Large-scale radiolabeling of Macropa-PEG$_{0,4,8}$-YS5 conjugates with Ac-225 (4:1 ratios). Radiochemical purity was monitored by radio-iTLC-SG using 10 mM EDTA pH=5.5 as an eluent after purification via centrifugal filtration. All radio-iTLC's were scanned after 24h equilibrium was reached.

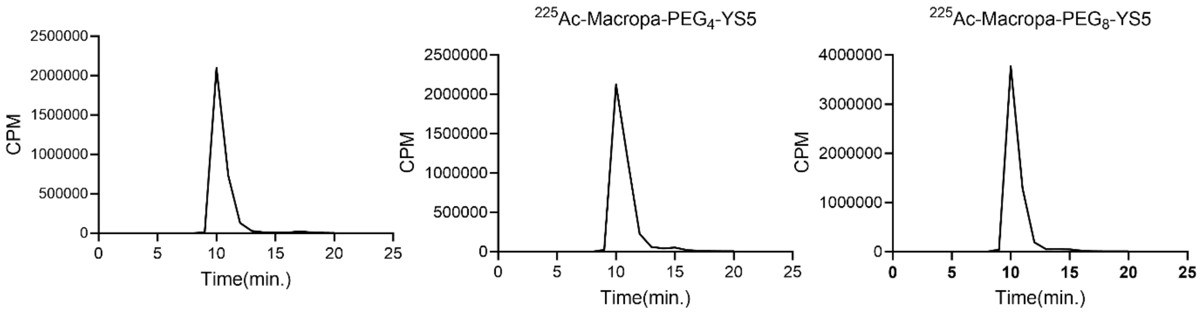

FIGURE S14. Size exclusion chromatograms of ^{225}Ac-Macropa-PEG$_{0,4,8}$-YS5(Left to right) after centrifugal filtration purification.

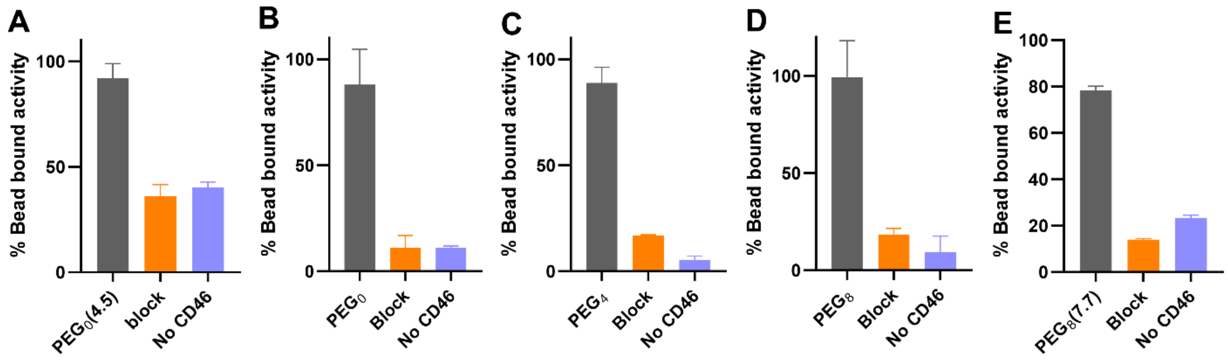

Figure S15. Magnetic bead-based assay for radiolabeled Immunoconjugates A) ^{225}Ac-Macropa-PEG$_0$(4.5)-YS5 (4.5 chelators), B) ^{225}Ac-Macropa-PEG$_0$-YS5, C) ^{225}Ac-Macropa-PEG$_4$-YS5, D) ^{225}Ac-Macropa-PEG$_8$-YS5, E) ^{225}Ac-Macropa-PEG$_8$(7.7)-YS5 (7.7 chelators);

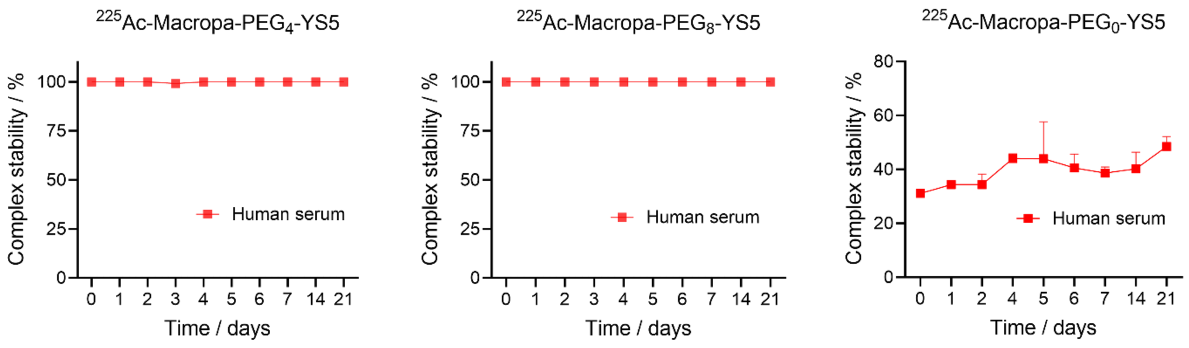

Figure S16. Stability studies of ^{225}Ac-Macropa-PEG$_4$-YS5, ^{225}Ac-Macropa-PEG$_8$-YS5, and ^{225}Ac-Macropa-PEG$_0$-YS5 in human serum until 21 days.

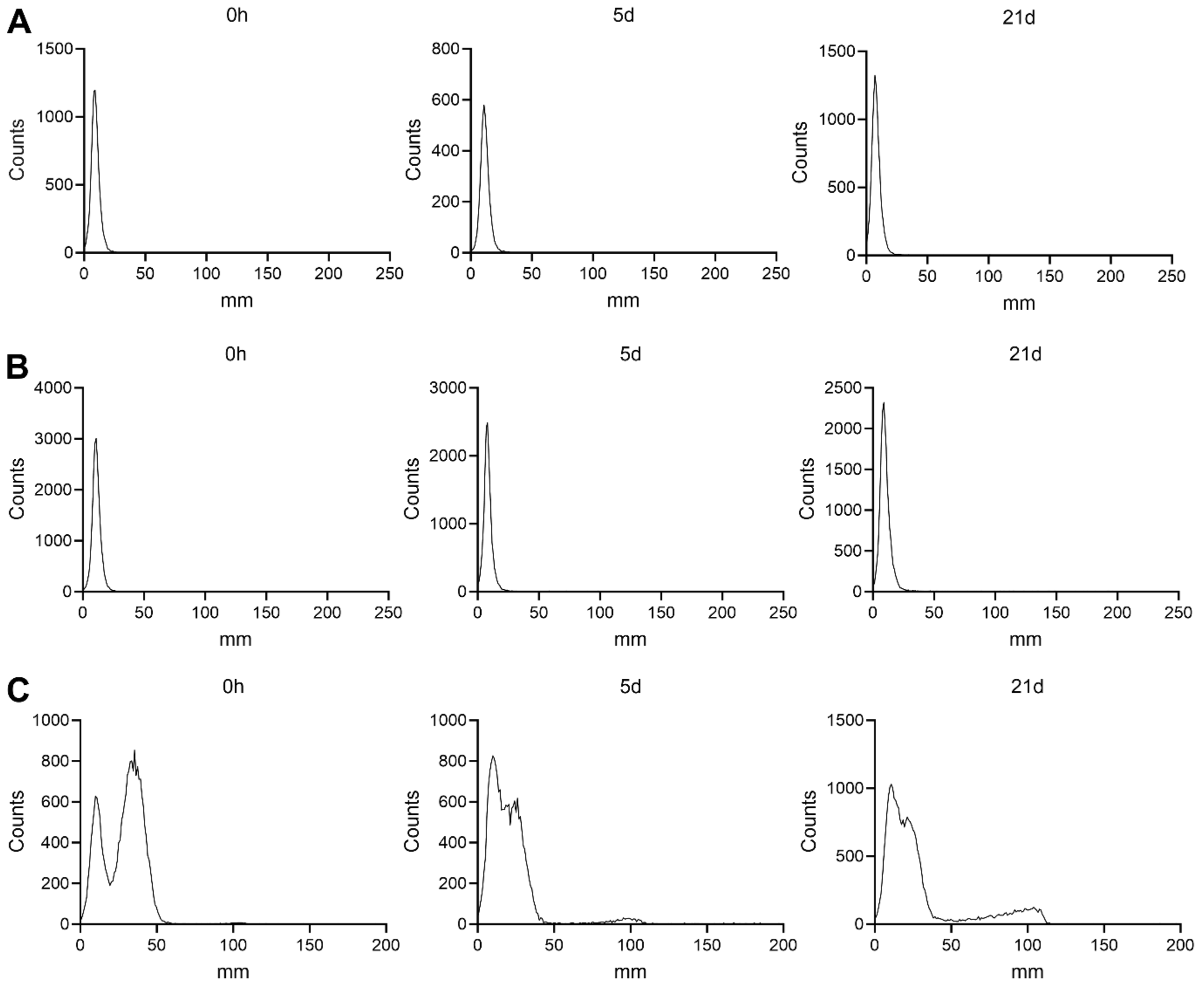

FIGURE S17. Radio iTLC's for selected time points 0, 5, and 21 days of ^{225}Ac-Macropa-PEG$_4$-YS5 (A), ^{225}Ac-Macropa-PEG$_8$-YS5 (B), and ^{225}Ac-Macropa-PEG$_0$-YS5 (C) in Human serum; All radio-iTLC's were scanned after 24 h equilibrium was reached.

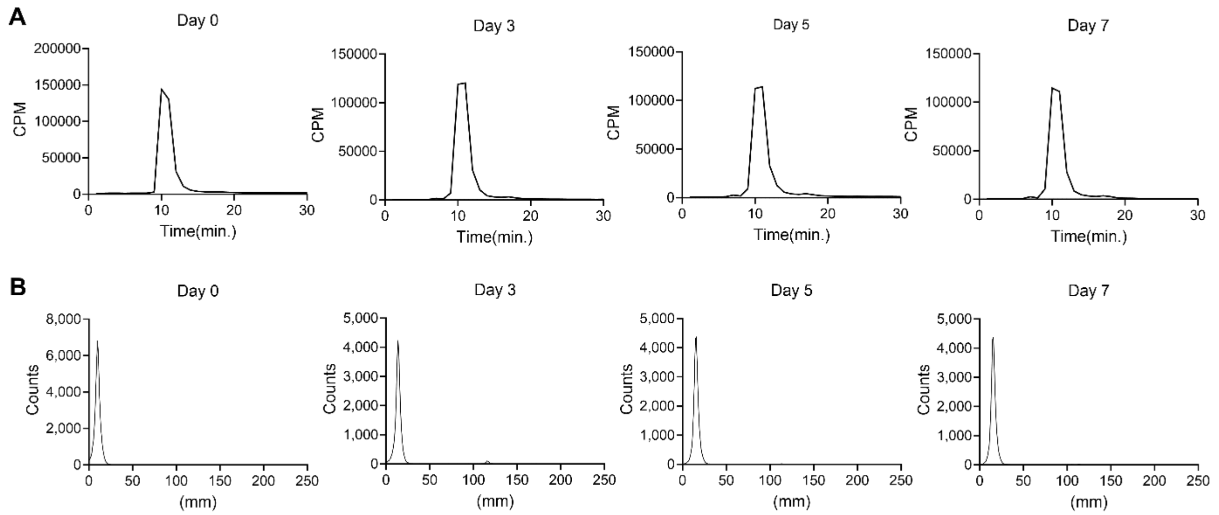

Figure S18. The stability of ^{225}Ac-Macropa-PEG$_4$-YS5 was monitored by SEC after incubating at 37 ^{o}C in 1% Human serum albumin (HSA) in Saline at 0, 3, 5, and 7 days (a); Radio iTLC's at the same time points (b). All radio-iTLC’s were scanned after 24 h equilibrium was reached.

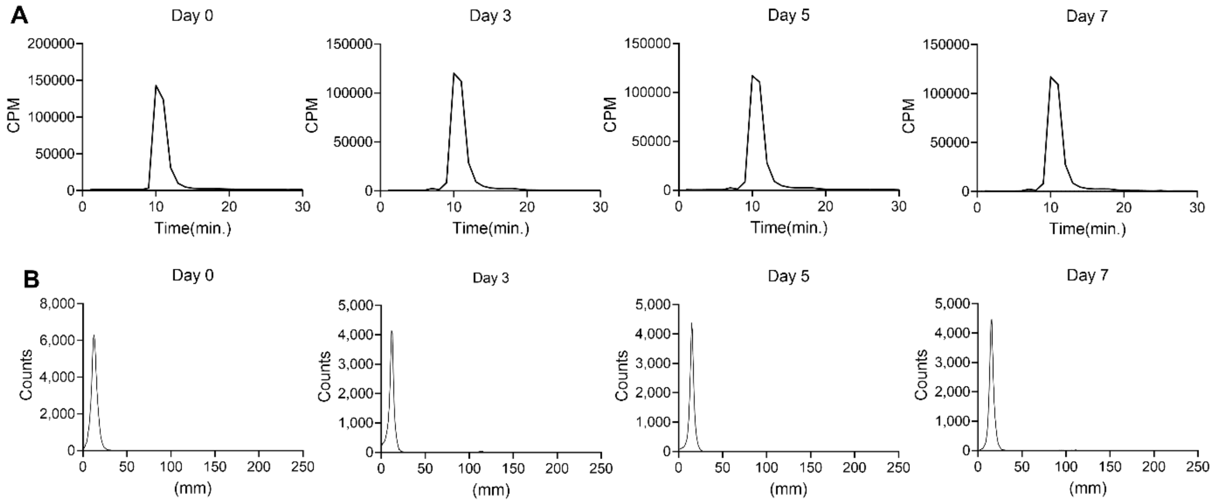

Figure S19. The stability of ^{225}Ac-Macropa-PEG$_8$-YS5 was monitored by SEC after incubating at 37 ^{o}C in 1% Human serum albumin in Saline at 0, 3, 5, and 7 days (A); Radio iTLC's at the same time points (B). All radio-iTLC’s were scanned after 24 h equilibrium was reached.

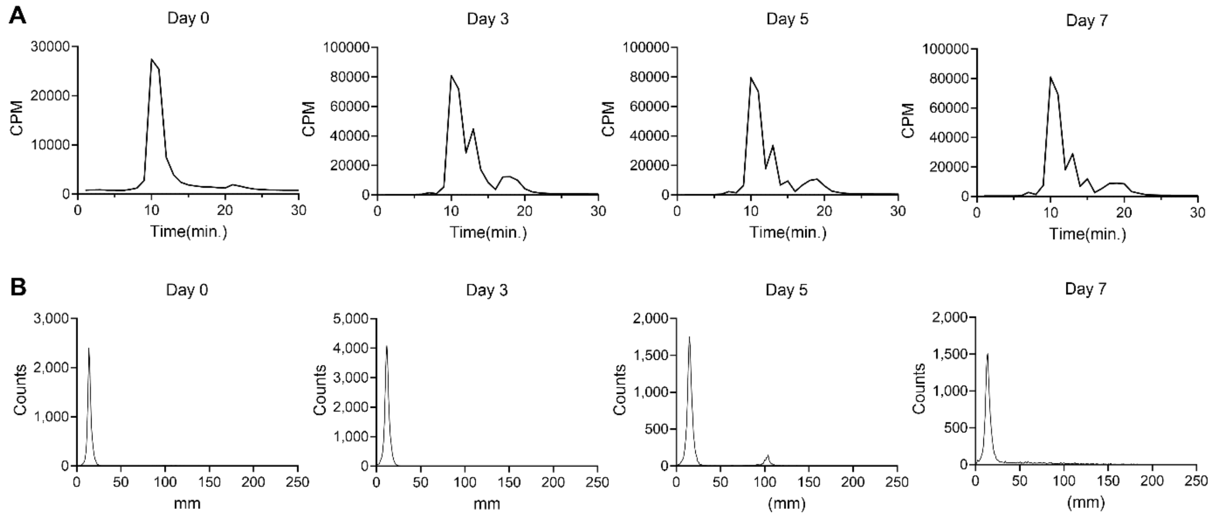

Figure S20. The stability of ^{225}Ac-Macropa-PEG$_0$-YS5 was monitored by SEC after incubating at 37 ^{o}C in 1% Human serum albumin in Saline at 0, 3, 5, and 7 days (A); Radio iTLC' s at the same time points (B). All radio-iTLC’s were scanned after 24 h equilibrium was reached.

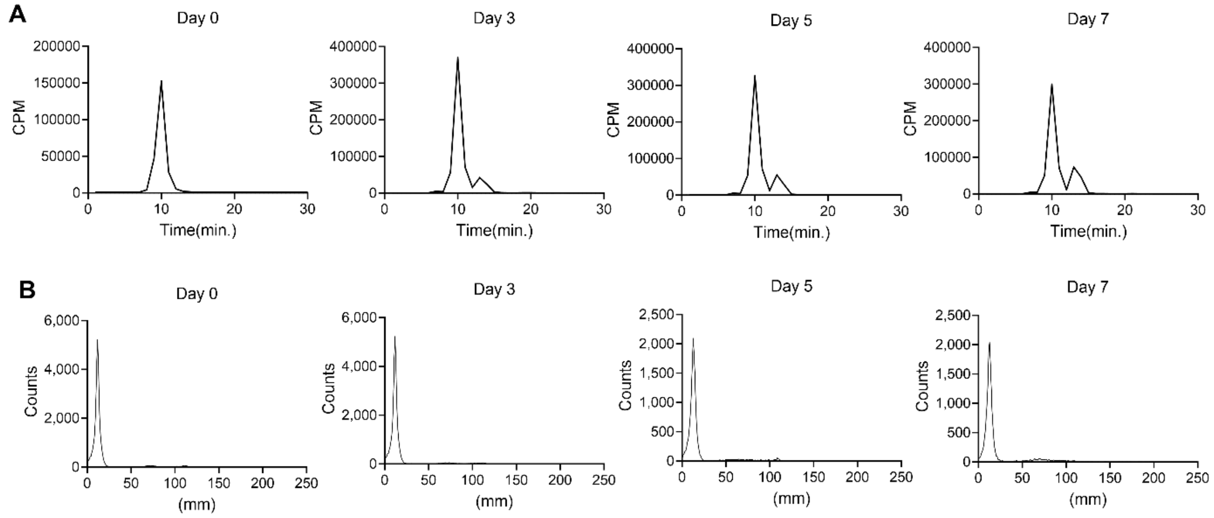

Figure S21. The stability of ^{225}Ac-DOTA-YS5 was monitored by SEC after incubating at 37 ^{o}C in 1% Human serum albumin in Saline at 0, 3, 5, and 7 days (a); Radio iTLC' s at the same time points (b). All radio-iTLC’s were scanned after 24 h equilibrium was reached.

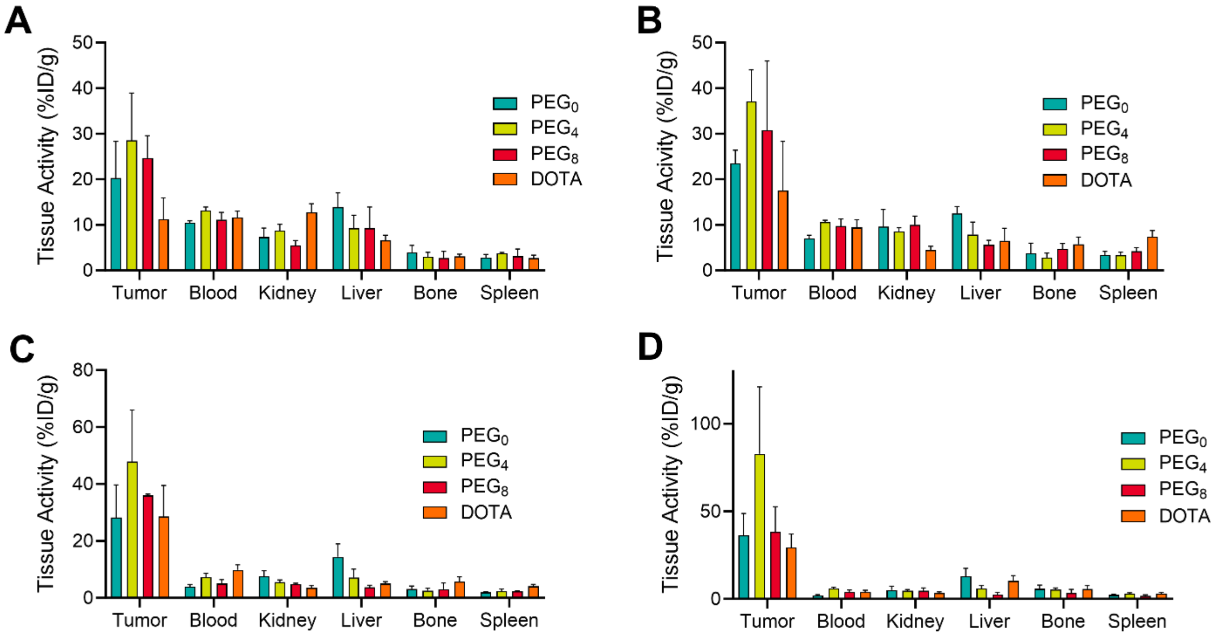

Figure 22. Ex-vivo biodistribution analysis of ^{225}Ac-Macropa-PEG$_{0/4/8}$-YS5 and ^{225}Ac-DOTA-YS5 in 22Rv1 xenografts over 7 days. Day 1 (A), Day 2 (B), Day 4 (C), Day 7 (D). ^{225}Ac-DOTA-YS5 biodistribution data was reproduced. Adapted with permission from [22], copyright 2023 American Association for Cancer Research.

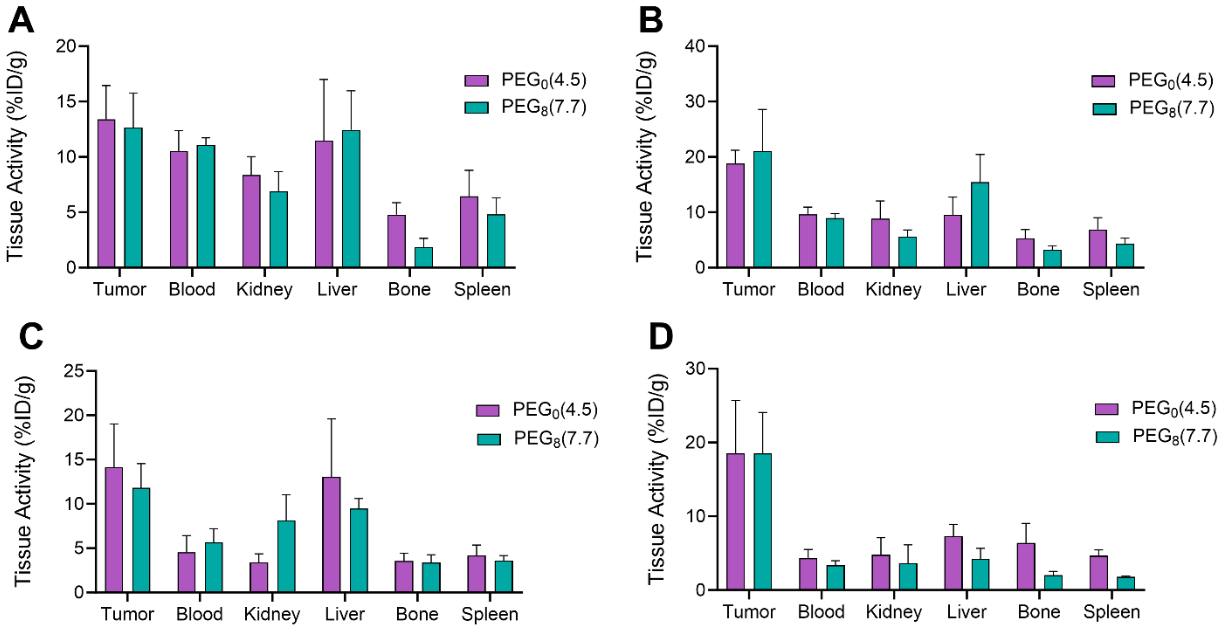

Figure S23. Biodistribution of ^{225}Ac-Macropa-PEG$_0$(4.5)-YS5, and ^{225}Ac-Macropa-PEG$_8$(7.7)-YS5 in 22Rv1 xenografts at different time points day 1 (A), day 2 (B), day 4 (C), day 7 (D).

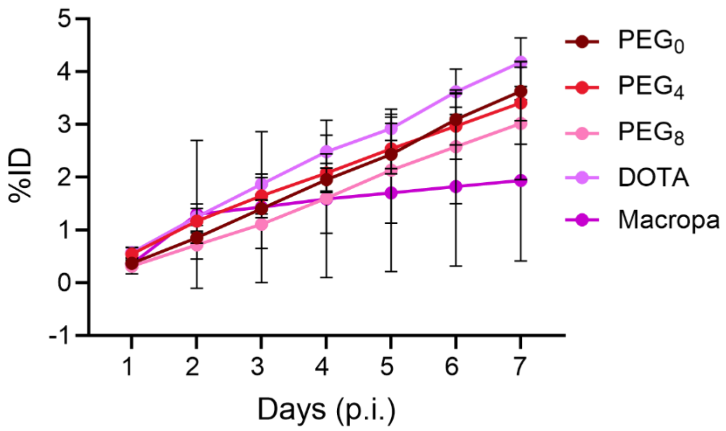

Figure S24 Metabolic analysis of ^{225}Ac-Macropa-PEG$_{0/4/8}$-YS5 and ^{225}Ac-DOTA-YS5 in healthy nude mice over 7 days, %ID from fecal elimination.

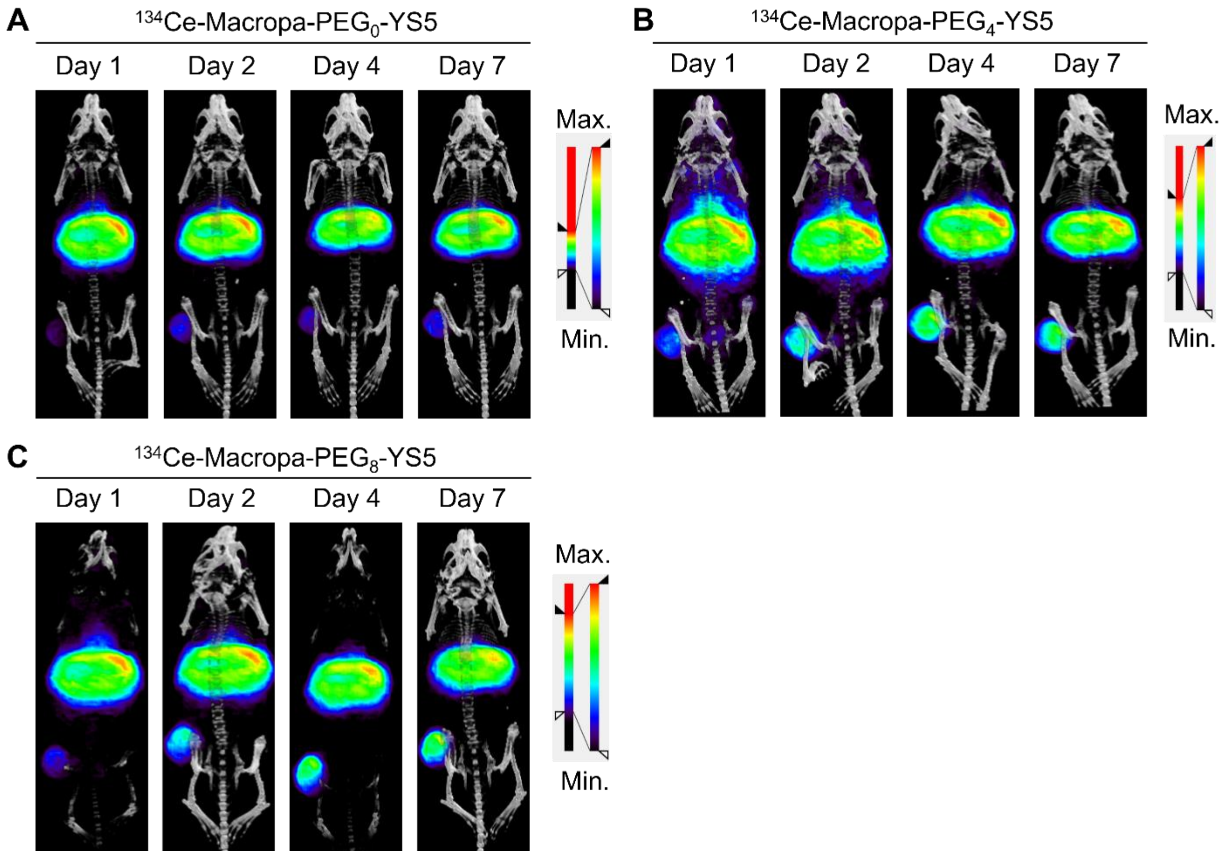

Figure S25. Maximum intensity projection (MIP) µPET/CT images of ^{134}Ce-Macropa-YS5 agents at 1, 2, 4, and 7 days to a mouse-bearing subcutaneous 22Rv1 xenografts after administering the A) ^{134}Ce-Macropa-PEG$_0$-YS5. B) ^{134}Ce-Macropa-PEG$_4$-YS5. C) ^{134}Ce-Macropa-PEG$_8$-YS5.via tail vein injection (n = 4 mice for each group).

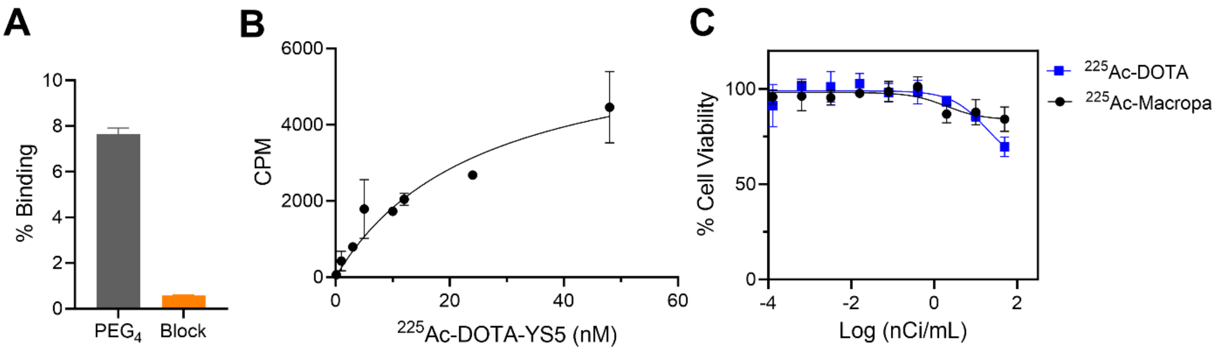

Figure S26. A) Cell binding of ^{225}Ac-Macropa-PEG$_4$-YS5 on 22Rv1 cells, with or without blocking with blocking of cold YS5 (n=3). B) Saturation binding assay of ^{225}Ac-DOTA-YS5 in 22Rv1 cells, indicating a K_d of 25.4±8.2 nM (n=3). C) Activity-dependent cell viability assay for a non-targeted control ^{225}Ac-DOTA and Macropa; A mild cell death was observed at higher concentrations.

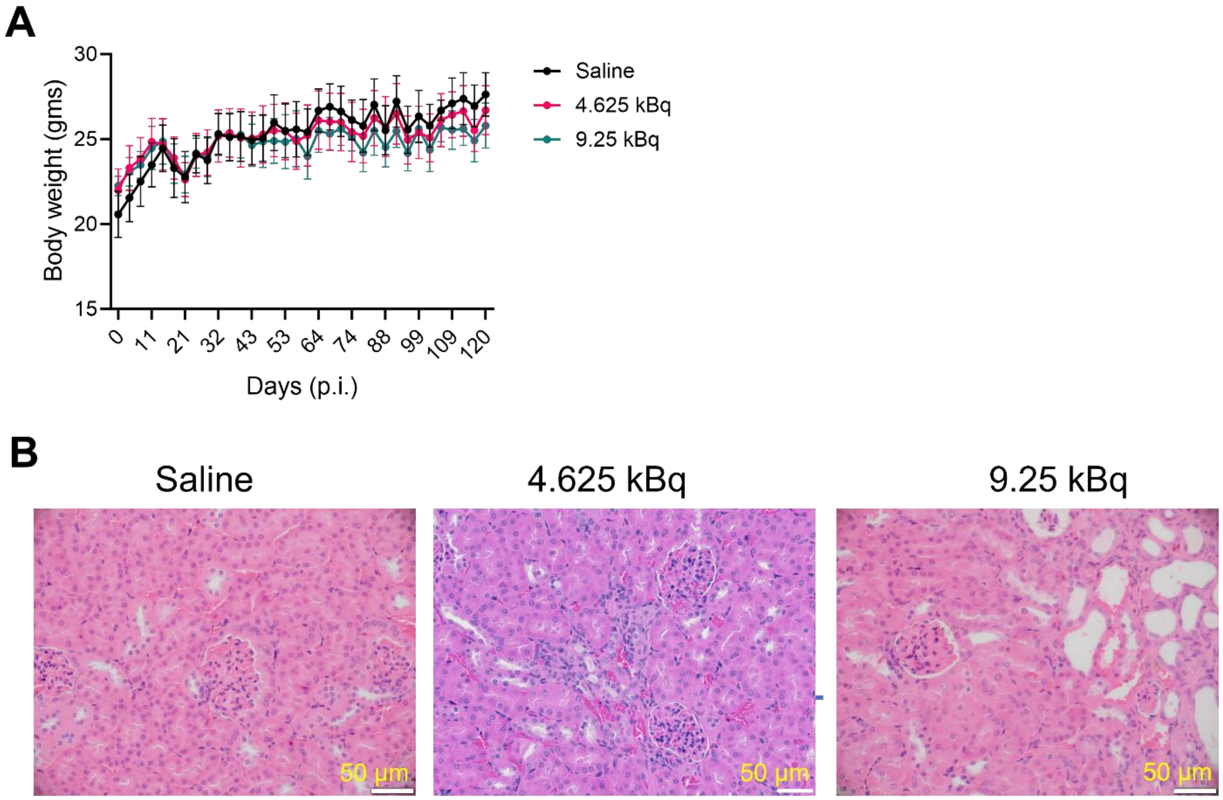

Figure S27. Chronic toxicity studies of ^{225}Ac-Macropa-PEG$_4$-YS5 versus saline as a control in athymic nude mice (*n* = 5). A) Body weights were measured twice weekly, with no weight loss observed. B) Hematoxylin and Eosin (H&E) staining of mouse kidney mouse injected with 4.625 and 9.25 kBq doses, An increased glomerular fibrin deposition and reduced capillaries in numerous glomeruli.

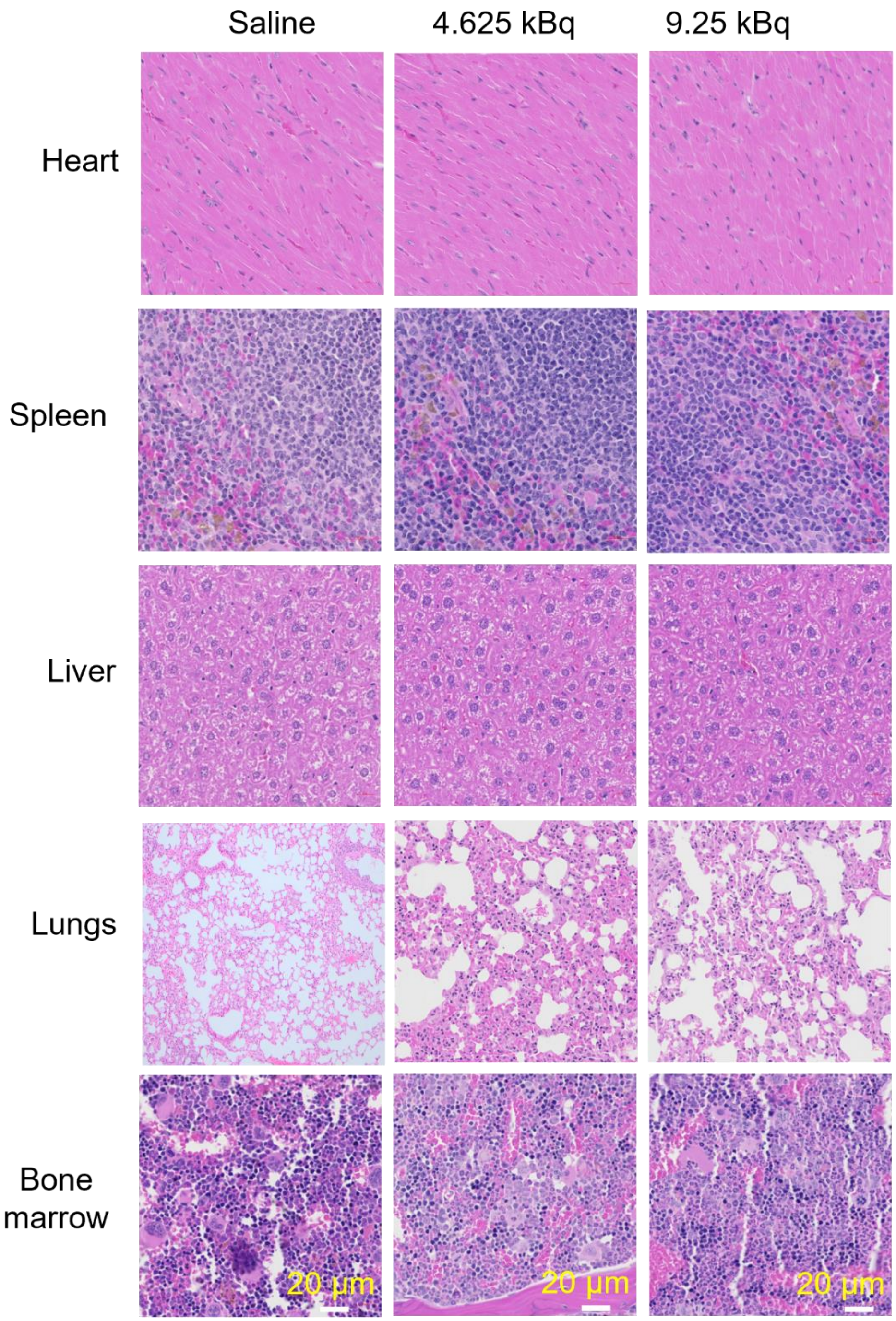

Figure S28. Chronic toxicity study, H&E staining of mouse organs heart, spleen, liver, lungs, and bone marrow injected with 4.625 and 9.25 kBq doses and saline control. No significant damage to the normal organs treated with 4.625 and 9.25 kBq were seen versus saline control; Scale bar: 20 µm

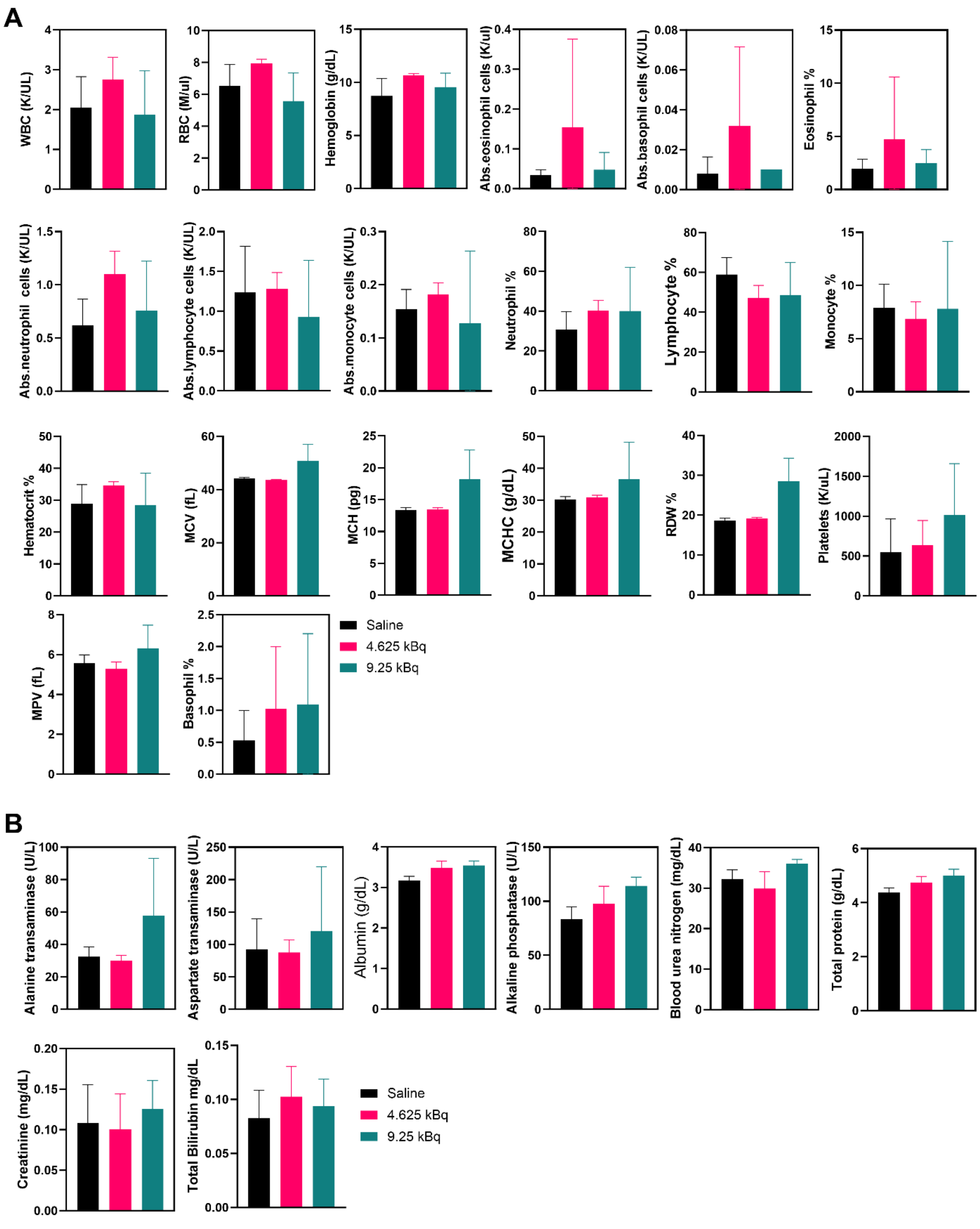

Figure S29. Completed blood analysis (A) and liver and kidney function test (B) for ^{225}Ac-Macropa-PEG$_4$-YS5 doses of 4.625, 9.25 kBq, and saline control cohort at day 125, showing no significant changes in treated versus saline control cohorts (n = 5 for each group).

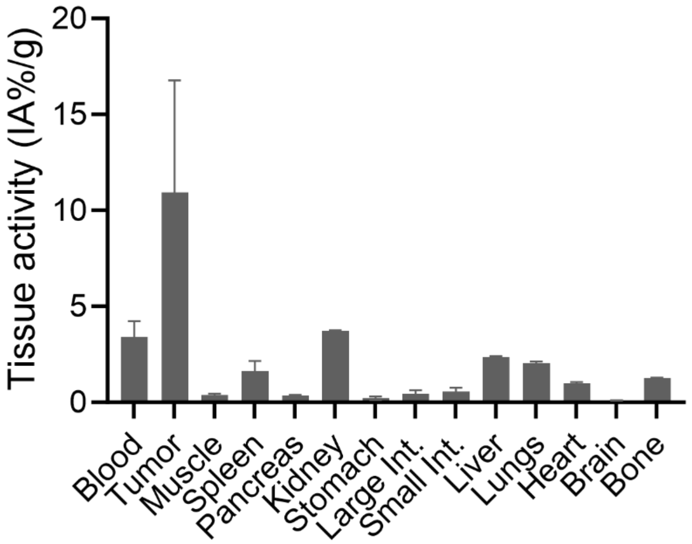

Figure S30. Ex-vivo biodistribution at day 4 after PET imaging with ^{89}Zr-DFO-YS5 for group 1 mice treated with fractionation dose ^{225}Ac-Macropa-PEG$_4$-YS5 (3 x 4.625 kBq) in 22Rv1 xenografts.

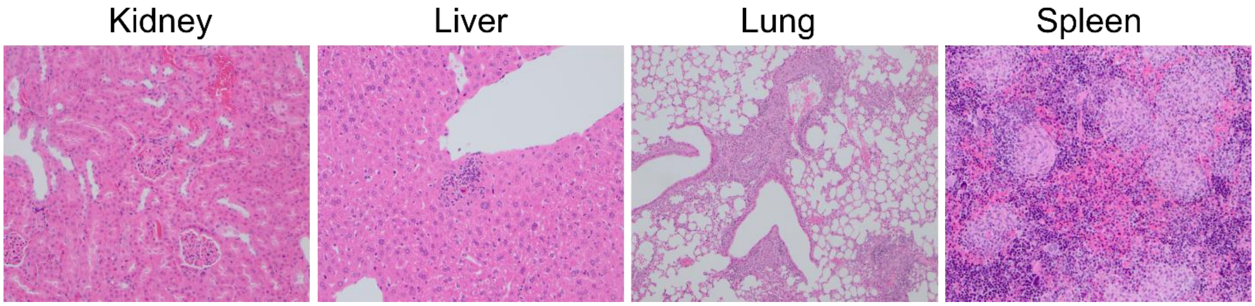

Figure S31. H&E staining of mouse kidney after 3 x 4.625 kBq fractionated treatments with ^{225}Ac-Macropa-PEG$_4$-YS5. Mild to moderate kidney toxicity was observed; one mouse found abnormal findings, centrizonal inflammation and focal centrizonal necrosis in the liver, the perivascular and peribronchiolar fibrosis and inflammation in the lungs, with focal acute inflammation, and the spleen showed scattered histiocytic aggregates and the other 2 mice were normal (n=3, group 2).

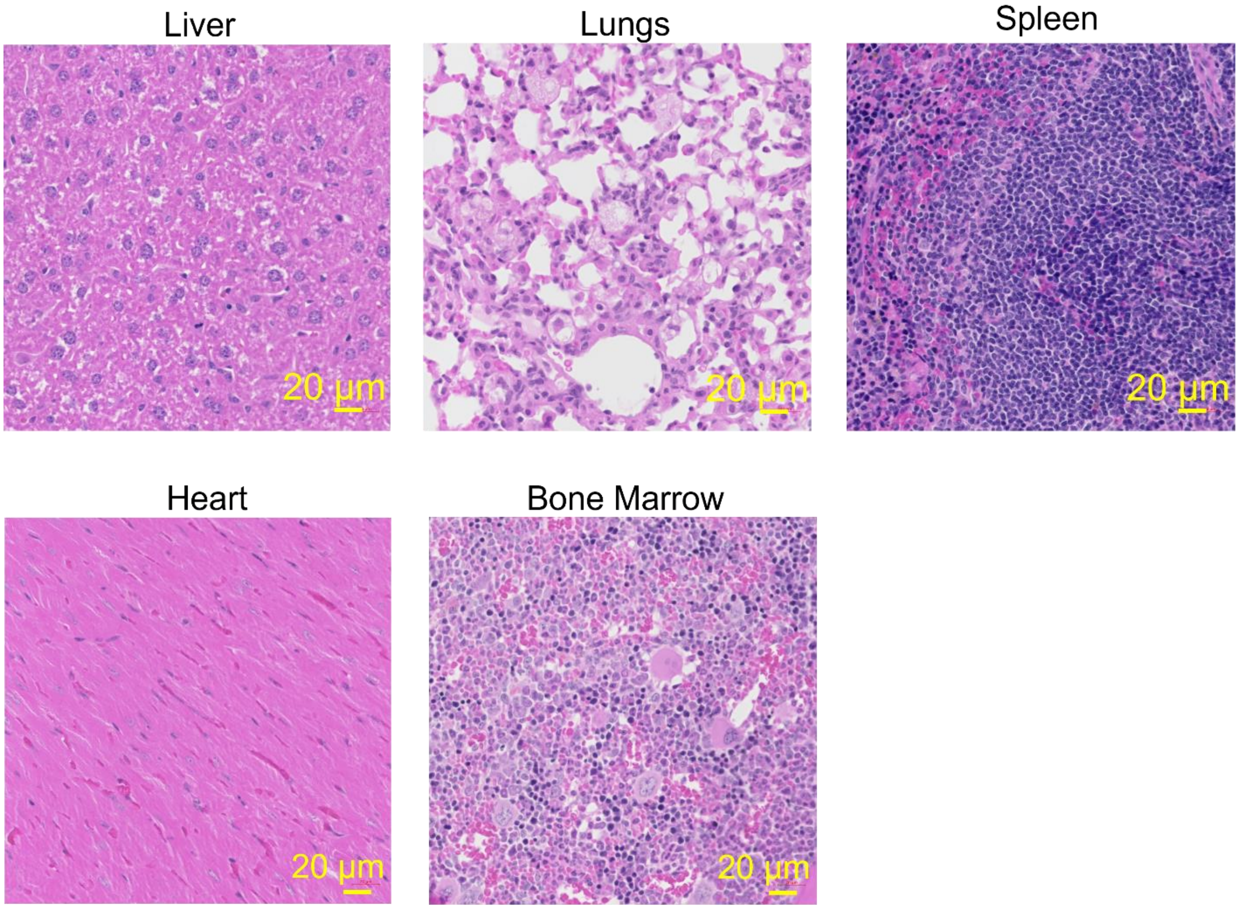

Figure S32. H&E staining of mouse organs heart, spleen, liver, lungs, and bone marrow for fractionation (3 x 4.625 kBq) dose of ^{225}Ac-Macropa-PEG$_4$-YS5, showing no damage (n=3); one mouse showed abnormal findings, with centrizonal inflammation and focal centrizonal necrosis in the liver, perivascular and peribronchiolar fibrosis and inflammation in the lungs, with focal acute inflammation, and scattered histiocytic aggregates in the spleen. The other 2 mice were normal. Scale bar: 20 µm

3. Supplementary tables

Table S1: Number of chelators per antibody YS5 after conjugation with various Macropa-NCS and Macropa-$PEG_{4/8}$-TFP esters equivalents. DOTA-YS5 has 8.7 chelators per antibody (*5*)

Conjugate Name	Chelator Eq.	Chelator ratios/YS5	No. of trials
Macropa-PEG_0-YS5	20	4.5	1
	15	0.55	1
Macropa-PEG_4-YS5	15	2.2	1
	7.5	0.91/0.96	2
Macropa-PEG_8-YS5	15	7.76	1
	7.5	0.96/0.86	2

Table S2: Radiolabeling of Macropa-$PEG_{0,4,8}$-YS5 and DOTA-YS5 (Ab) with $^{225}Ac(NO_3)_3$ in 0.2M HCl, *(1:4 ratio of ^{225}Ac $_{VS}$YS5); (#) = N*umber of chelators per YS5 antibody. *One reaction was purified only; BP=Before purification; AP=After purification.

Conjugate Name	RCY BP (%)	RCP AP (%)	Isolated Yield (%)	SA (µCi/µg)	No. of entry
Macropa-PEG_0(4.5)-YS5	96.01±1.16	98.54±0.34	80±0.0	0.2±0.0	3
Macropa-PEG_0(0.56)-YS5	94.76±2.46	94.85±2.1	33.1±9.0	0.08±0.02	3
Macropa-PEG_4(0.91)-YS5	95.53±1.1	96.89±3.2	70.11±7.12	0.17±0.017	6
Macropa-PEG_4(2.2)-YS5	94.85	96.79	68.33	0.17	1¶
Macropa-PEG_8(7.7)-YS5	97.74±1.6	100	67.5	0.17	3*
Macropa-PEG_8(0.96)-YS5	97.20±1.05	97.12±2.19	62.4±16.36	0.16±0.04	3
DOTA (8.7)-YS5	75.07±6.13	94.4±1.72	35.12±9.35	0.09±0.024	4

Table S3: Percentage of stability for ^{225}Ac-Macropa-$PEG_{0/4/8}$-YS5 and ^{225}Ac-DOTA-YS5 in 1% HSA in saline at day 0, 3, 5, and 7 calculated from SEC chromatograms.

Days	^{225}Ac-Macropa-PEG_0-YS5	^{225}Ac-Macropa-PEG_4-YS5	^{225}Ac-Macropa-PEG_8-YS5	^{225}Ac-DOTA-YS5
Day 0	100	100	100	100
Day 3	29.4	100	100	88.63
Day 5	32.3	100	100	83.06
Day 7	38.2	100	100	84.76

Table S4: Ex vivo biodistribution of ^{225}Ac-Macropa-PEG_0-YS5 in mouse bearing 22Rv1 xenografts subcutaneously at day 1, 2, 4 and 7 post-injections.

Organs	^{225}Ac-Macropa-PEG$_0$(4.5)-YS5			
	Day 1 (n=5)	Day 2 (n=5)	Day 4 (n=5)	Day 7 (n=5)
Tumor	13.4 ± 3.40	18.8 ± 2.42	14.14 ± 4.87	18.53 ± 7.17
Blood	10.53 ± 1.86	9.63 ± 1.31	4.56 ± 1.87	4.32 ± 1.20
Muscle	1.04 ± 0.11	1.15 ± 0.27	1.09 ± 0.22	0.92 ± 0.19
Bone	4.79 ± 1.09	5.3 ± 1.58	3.55 ± 0.88	6.38 ± 2.66
Kidney	8.37 ± 1.66	8.84 ± 3.20	3.4 ± 0.96	4.8 ± 2.31
Liver	11.45 ± 5.54	9.56 ± 3.20	13.03 ± 6.56	7.3 ± 1.62
Large	1.62 ± 0.42	1.98 ± 0.43	1.11 ± 0.31	1.3 ± 0.20
Small	1.77 ± 0.55	2.19 ± 0.32	1.19 ± 0.26	1.42 ± 0.35
Heart	5.04 ± 0.94	5.32 ± 1.55	2.56 ± 0.52	2.71 ± 0.45
Lungs	5.03 ± 0.98	4.85 ± 0.95	2.98 ± 0.80	3.47 ± 0.86
Brain	0.68 ± 0.38	0.41 ± 0.04	0.54 ± 0.20	0.51 ± 0.10
Stomach	0.83 ± 0.13	0.61 ± 0.22	0.37 ± 0.13	0.43 ± 0.10
Spleen	6.46 ± 2.33	6.91 ± 2.09	4.17 ± 1.20	4.67 ± 0.81
Pancreas	1.47 ± 0.19	1.55 ± 0.24	1.4 ± 0.24	1.62 ± 0.22
**Organs**	**^{225}Ac-Macropa-PEG$_0$-YS5**			
	Day 1 (n=4)	Day 2 (n=4)	Day 4 (n=4)	Day 7 (n=5)
Tumor	20.31 ± 8.02	23.49 ± 2.89	28.24 ± 11.44	36.39 ± 12.4
Blood	10.43 ± 0.49	7.04 ± 0.71	3.94 ± 0.78	2.01 ± 0.43
Muscle	0.97 ± 0.18	0.88 ± 0.1	0.69 ± 0.44	0.83 ± 0.26
Bone	3.97 ± 1.54	3.79 ± 2.2	3.16 ± 0.99	5.77 ± 2.09
Kidney	7.31 ± 2.01	9.59 ± 3.82	7.62 ± 1.92	5.18 ± 2.06
Liver	13.87 ± 3.16	12.51 ± 1.51	14.45 ± 4.5	12.97 ± 4.5
Large	1.11 ± 0.07	1.52 ± 0.28	1.18 ± 0.48	1.17 ± 0.14
Small	1.72 ± 0.24	1.52 ± 0.3	1.08 ± 0.23	1.21 ± 0.22
Heart	4.26 ± 0.62	3.23 ± 0.49	2.44 ± 0.71	1.74 ± 0.22
Lungs	5.83 ± 1.03	4.62 ± 1.48	3.37 ± 1.1	2.32 ± 0.26
Brain	0.40 ± 0.06	0.06 ± 0.06	0.23 ± 0.03	0.29 ± 0.06
Stomach	1.01 ± 0.29	1.03 ± 0.43	0.47 ± 0.14	0.52 ± 0.14
Spleen	2.83 ± 0.69	3.38 ± 0.81	1.96 ± 0.16	2.25 ± 0.5
Pancreas	1.17 ± 0.13	1.1 ± 0.06	0.8 ± 0.09	0.93 ± 0.14

Table S5: Ex vivo biodistribution of ^{225}Ac-Macropa-PEG$_8$-YS5 in mice bearing 22Rv1 xenografts subcutaneously at day 1, 2, 4, and 7 post-injections.

**Organs**	**^{225}Ac-Macropa-PEG$_8$-YS5**			
	Day 1 (n=5)	Day 2 (n=4)	Day 4 (n=4)	Day 7 (n=4)
Tumor	24.64 ± 4.92	30.79 ± 15.18	35.98 ± 0.45	38.15 ± 14.41
Blood	11.14 ± 1.59	9.75 ± 1.58	5.02 ± 1.38	3.86 ± 1.33
Muscle	1.02 ± 0.10	1.38 ± 0.51	0.86 ± 0.13	0.74 ± 0.07
Bone	2.74 ± 1.44	4.76 ± 1.18	3.01 ± 2.24	3.33 ± 2.16
Kidney	5.43 ± 1.14	10.03 ± 1.92	4.83 ± 0.36	4.56 ± 1.63
Liver	9.29 ± 4.66	5.63 ± 0.99	3.68 ± 0.79	2.56 ± 1.19
Large	1.6 ± 0.12	1.86 ± 0.36	1 ± 0.18	0.92 ± 0.39
Small	1.73 ± 0.34	2.21 ± 0.72	1.04 ± 0.18	1.15 ± 0.35
Heart	4 ± 1.13	4.27 ± 0.45	2.54 ± 0.40	2.12 ± 0.62
Lungs	4.83 ± 1.01	5.51 ± 1.17	2.79 ± 0.96	2.91 ± 0.80
Brain	0.41 ± 0.13	0.49 ± 0.14	0.36 ± 0.14	0.37 ± 0.10
Stomach	0.59 ± 0.15	0.65 ± 0.21	0.49 ± 0.12	0.36 ± 0.09
Spleen	3.13 ± 1.56	4.17 ± 0.84	2.32 ± 0.18	1.83 ± 0.54
Pancreas	1.27 ± 0.20	1.75 ± 0.49	0.99 ± 0.25	1.1 ± 0.14
	**^{225}Ac-Macropa-PEG$_8$(7.7)-YS5**			
**Organs**	Day 1 (n=4)	Day 2 (n=4)	Day 4 (n=3)	Day 7 (n=4)
Tumor	12.65 ± 3.10	21.06 ± 7.51	11.82 ± 2.72	18.51 ± 5.55
Blood	11.08 ± 0.64	8.95 ± 0.83	5.62 ± 1.57	3.39 ± 0.59
Muscle	0.94.02 ± 0.11	1.04 ± 0.42	1.40 ± 0.52	1.12 ± 0.50
Bone	1.84 ± 0.80	3.22 ± 0.66	3.37 ± 0.88	2.04 ± 0.48
Kidney	6.89 ± 1.77	5.62 ± 1.20	8.14 ± 2.89	3.63 ± 2.50
Liver	12.37 ± 3.49	15.43 ± 5.03	9.47 ± 1.14	4.22 ± 1.43
Large	1.71± 0.43	1.36± 0.89	1.72± 0.47	0.82 ± 0.30
Small	1.86± 0.41	1.52 ± 0.78	1.31 ± 0.60	1.07 ± 0.42
Heart	3.53± 0.35	3.26 ± 0.67	3.14 ± 0.77	2.04 ± 0.29
Lungs	6.09 ± 0.22	3.79 ± 1.04	4.95 ± 2.85	2.52 ± 0.45
Brain	0.30 ± 0.08	0.34 ± 0.14	0.42 ± 0.12	0.38 ± 0.08
Stomach	0.66 ± 0.22	0.81 ± 0.41	0.50 ± 0.07	0.27 ± 0.12
Spleen	4.71 ± 1.30	4.29 ± 1.04	3.59 ±0 57	1.77 ± 0.14
Pancreas	1.22 ± 0.15	1.04 ± 0.47	1.16 ± 0.31	1.36 ± 0.37

Table S6: Ex vivo biodistribution of ^{225}Ac-Macropa-PEG$_4$-YS5 in mice bearing 22Rv1 xenografts subcutaneously at day 1, 2, 4, and 7 post-injection.

**Organs**	**^{225}Ac-Macropa-PEG$_4$-YS5**			
	Day 1 (n=4)	Day 2 (n=4)	Day 4 (n=4)	Day 7 (n=5)
Tumor	28.54 ± 10.40	37.09 ± 6.99	47.85 ± 18.18	82.82 ± 38.27
Blood	13.17 ± 0.78	10.63 ± 0.37	7.25 ± 1.38	5.87 ± 0.83
Muscle	1.16 ± 0.22	1.19 ± 0.21	0.8 ± 0.15	0.88 ± 0.09
Bone	3.03 ± 0.97	2.82 ± 0.99	2.56 ± 0.86	5.38 ± 0.84
Kidney	8.79 ± 1.37	8.58 ± 0.79	5.54 ± 0.79	4.67 ± 0.73
Liver	9.28 ± 2.82	7.92 ± 2.69	7.18 ± 2.92	6.04 ± 1.72
Large [illegible]	2.13 ± 1.10	1.57 ± 0.23	1.23 ± 0.25	1.11 ± 0.24
Small [illegible]	1.81 ± 0.54	1.69 ± 0.40	1.14 ± 0.15	1.33 ± 0.42
Heart	4.84 ± 0.94	3.53 ± 0.47	2.78 ± 0.72	2.55 ± 0.39
Lungs	7.46 ± 2.36	6.66 ± 1.48	4.29 ± 0.58	5.59 ± 2.16
Brain	0.29 ± 0.10	0.31 ± 0.07	0.28 ± 0.08	0.32 ± 0.07
Stomach	0.95 ± 0.31	0.88 ± 0.30	0.41 ± 0.20	0.61 ± 0.20
Spleen	3.78 ± 0.14	3.36 ± 0.68	2.34 ± 0.84	3.12 ± 0.42
Pancreas	1.47 ± 0.10	1.05 ± 0.05	0.98 ± 0.23	1.06 ± 0.13

Table S7: Statistical analysis for tumor uptakes ^{225}Ac-Macropa-PEG$_4$-YS5 versus other radio immunoconjugates. Two-way ANOVA is used for p-value calculations * P ≤ 0.05, ** P ≤ 0.01, ns. = non-significant

**Days**	**PEG$_0$(4.5)**	**PEG$_0$**	**PEG$_8$**	**PEG$_8$(7.7)**	**DOTA**
Day 1	****	**	ns	****	****
Day 2	****	****	ns	****	****
Day 4	****	****	**	****	****
Day 7	****	****	****	****	****

Table S8: Tumor to blood, muscle, liver, and kidney ratios were calculated from the ex vivo biodistribution data.

	**Tumor-to-blood**					
**Days**	**PEG_0(4.5)**	**PEG_0**	**PEG_4**	**PEG_8**	**PEG_8(7.7)**	**DOTA (8.7)**
1	1.26± 0.067	1.95 ± 0.79	2.18 ± 0.84	2.21 ± 0.38	1.15 ±0.32	0.95 ±0.36
2	1.95 ± 0.14	3.34 ± 0.34	3.49 ± 0.67	3.040 ± 1.246	2.36 ±0.84	1.85 ±1.02
4	3.32 ± 1.17	7.61 ±3.66	6.71 ±2.37	7.58 ± 2.12	2.26 ±0.93	3.06 ±1.47
7	4.19 ± 0.70	17.93 ± 4.17	13.70 ±4.93	10.57 ± 1.99	5.57 ±1.90	7.89 ±3.33
	**Tumor-to-Muscle**					
**Days**	**PEG_0(4.5)**	**PEG_0**	**PEG_4**	**PEG_8**	**PEG_8(7.7)**	**DOTA (8.7)**
1	12.75 ± 1.55	21.85 ± 9.61	24.13 ± 5.30	24.22± 4.75	13.80 ±5.34	27.07 ±11.29
2	17.37 ± 6.29	26.76 ± 2.72	31.39 ± 4.98	21.13 ± 4.75	20.61 ±5.65	16.66 ±10.81
4	13.94 ± 7.59	44.81 ±14.31	61.63 ±25.35	48.39 ± 15.77	9.24 ±4.19	64.92 ±42.61
7	20.83 ± 9.80	43.68 ± 7.71	95.67 ±46.38	47.55 ± 13.55	18.28 ±8.59	81.96 ±41.61
	**Tumor-to-Liver**					
**Days**	**PEG_0(4.5)**	**PEG_0**	**PEG_4**	**PEG_8**	**PEG_8(7.7)**	**DOTA (8.7)**
1	1.39± 0.619	1.61 ± 0.90	3.38 ± 1.76	2.93±0.80	1.10±0.47	2.74 ±1.83
2	2.2 ± 0.95	1.89 ± 0.28	5.21 ± 2.10	5.74 ± 3.45	1.53 ±0.74	3.21 ±2.15
4	1.42 ± 1.04	2.24 ±1.51	7.56 ±4.65	3.67 ± 0.79	1.27 ±0.37	5.77 ±2.39
7	2.50 ± 0.80	2.91 ± 0.78	14.29 ±8.02	15.58 ±3.88	4.96 ±2.43	2.86 ±0.83
	**Tumor-to-Kidney**					
**Days**	**PEG_0(4.5)**	**PEG_0**	**PEG_4**	**PEG_8**	**PEG_8(7.7)**	**DOTA (8.7)**
1	1.62± 0.44	3.13 ± 2.03	3.24 ± 0.96	4.72 ± 1.47	1.85 ±0.21	6.26 ±1.25
2	2.32 ± 0.69	2.73 ± 0.98	4.39 ± 1.13	3.23 ±1.88	3.95 ±1.69	1.90 ±0.30
4	4.68 ± 2.55	3.63 ±0.84	8.75 ±3.20	7.47± 0.47	1.63 ±0.82	8.51 ±4.27
7	3.97 ± 1.04	7.39±1.92	18.70 ±11.29	8.60 ± 2.56	7.36 ±6.03	9.39 ±3.67

Table S9: Statistical analysis for tumor to background ratios, ^{225}Ac-Macropa-PEG_4(0.91)-YS5 versus other radio immunoconjugates. Two-way ANOVA is used for p-value calculations; ** P =0.0015, *** P =0.0001, **** P < 0.0001, ns = non-significant

	**PEG_0(4.5)**	**PEG_0**	**PEG_8**	**PEG_8(7.7)**	**DOTA**
Tumor-to-Blood	****	**	ns	****	**
Tumor-to-Muscle	****	****	***	****	ns
Tumor-to-Liver	****	****	ns	****	****
Tumor-to-Kidney	****	****	***	****	**

Table S10: Absorbed dose estimation for ^{225}Ac-Macropa-PEG$_{0/4/8}$-YS5 conjugates in organs and tumors. The highest dose was delivered to tumor tissue in comparison with other normal organs for all the conjugates.

**Organ**	**^{225}Ac-Macropa-PEG$_0$-YS5 (Sv)**	**^{225}Ac-Macropa-PEG$_4$-YS5 (Sv)**	**^{225}Ac-Macropa-PEG$_8$-YS5 (Sv)**
Tumor	10.42	29.07	21.55
Brain	0.45	0.46	0.43
Large Int	2.66	3.90	2.28
Small Intestine	2.55	3.79	2.26
Stomach Wall	6.75	7.05	4.85
Heart	3.44	4.75	3.02
Kidneys	49.03	16.98	6.77
Liver	0.22	0.33	0.25
Lungs	1.22	1.41	1.19
Pancreas	7.75	6.72	5.05
Skeleton	2.37	3.61	2.04
Spleen	2.04	1.55	0.70
Testes	2.37	3.61	2.04
Thyroid	2.37	3.61	2.04
Urine Blader	2.37	3.61	2.04
Total Body	3.22	4.07	2.35

Supplementary references:

1. Su Y, Liu Y, Behrens CR, Bidlingmaier S, Lee NK, Aggarwal R, *et al.* Targeting CD46 for both adenocarcinoma and neuroendocrine prostate cancer. JCI Insight. 2018; 3: e121497.
2. Thiele NA, Brown V, Kelly JM, Amor-Coarasa A, Jermilova U, MacMillan SN, *et al.* An eighteen-membered macrocyclic ligand for actinium-225 targeted alpha therapy. Angew Chem Int Ed. 2017; 56: 14712–17.
3. Wang S, Li J, Hua J, Su Y, Beckford-Vera DR, Zhao W, *et al.* Molecular imaging of prostate cancer targeting CD46 using immunoPET. Clin Cancer Res. 2021; 27: 1305–15.
4. Bobba KN, Bidkar AP, Meher N, Fong C, Wadhwa A, Dhrona S, *et al.* Evaluation of 134 Ce/ 134 La as a PET imaging theranostic pair for 225Ac α-radiotherapeutics J Nucl Med. 2023; 64: 1076-82.
